# One-pot redox cascade paired electrosynthesis of gamma-butyrolactone from furoic acid

**DOI:** 10.1038/s41467-024-45278-z

**Published:** 2024-02-07

**Authors:** Shengqin Liu, Yangxin Jin, Shuquan Huang, Qi Zhu, Shan Shao, Jason Chun-Ho Lam

**Affiliations:** 1 School of Energy and Environment, City University of Hong Kong, Kowloon Tong, Hong Kong SAR, 999077 China; 2 Faculty of Chemical Engineering, Kunming University of Science and Technology, Kunming, 650500 China; 3 State Key Laboratory of Marine Pollution, City University of Hong Kong, Kowloon Tong, Hong Kong SAR, 999077 China

**Keywords:** Sustainability, Electrocatalysis, Environmental chemistry

## Abstract

The catalytic valorisation of biomass to afford synthetically useful small molecules is essential for sustainable biorefinery processes. Herein, we present a mild cascaded electrochemical protocol for converting furoic acid, a common biomass-derived feedstock, into a versatile platform chemical, gamma-butyrolactone. In the platinum(+)|nickel(−) electrode paired undivided cell, furoic acid is electrochemically oxidised with 84.2% selectivity to 2(5*H*)-furanone, the olefin of which is then hydrogenated to yield gamma-butyrolactone with 98.5% selectivity. The final gamma-butyrolactone yield is 69.1% with 38.3% Faradaic efficiency and 80.1% carbon balance when the reaction is performed with 100 mM furoic acid at 80 °C at +2.0 V_Ag/AgCl_. Mechanistic investigation revealed the critical temperature and electrolyte pH conditions that maximise the production and protection of the key intermediate, furan radical, promoting its transition to 2(5*H*)-furanone rather than self-polymerising. The reaction is scalable, as 2.1 g of 98.1% pure gamma-butyrolactone is isolated through a simple solvent extraction.

## Abstract (web summary)

Paired electrolysis is challenging without membrane and redox mediators. The authors developed a one-pot, mediator-free, and scalable e-synthesis of γ-butyrolactone (GBL) from furoic acid with high yield and selectivity in an aqueous electrolyte.

## Introduction

The dwindling supply of fossil resources coupled with a growing interest in achieving sustainable goals has accelerated the development of biorefineries worldwide^1^. Biomass is the only renewable carbon source and thus presents the only opportunity to produce sustainable fuels and chemicals. Over the past few decades, biomass valorisation, i.e., the transformation of various lignocellulosic feedstocks into biofuels and bio-derived chemicals, has garnered considerable research attention^2^. Gamma-butyrolactone (GBL) is a prominent biorefinery compound due to its versatility; it is a non-toxic solvent and a chemical precursor applicable in various industries, including the spice, pharmaceutical, and perfume industries^3–6^. In 2022, the global market size for GBL reached USD 3614.07 million, and it is expected to exceed USD 4904.37 million by 2032^7^. In the conventional petroleum-based route for GBL production, benzene is oxidised to maleic anhydride under 1–3 bar of oxygen (O_2_) at 200–600 °C, and the maleic anhydride is hydrogenated to GBL under 6–8 MPa of hydrogen (H_2_) at 160–280 °C. Alternatively, acetylene is condensed with formaldehyde under 0.5–2.0 MPa of H_2_ at 90–110 °C to form 1,4-butanediol, which is dehydrogenatively ring closure at 180–300 °C to afford GBL^8–12^. Given the broad chemical and pharmaceutical applications of GBL, its synthesis from a renewable precursor is an attractive option for promoting sustainability in biorefineries. Thus, furanic compounds such as furoic acid (FA) and furfural (FAL) are valuable as starting materials due to their ready availability from hemicellulose, which is the world’s second-most abundant renewable polymer^13^.

In the biomass route, furanic precursors such as FAL and FA are oxidised to 2(5*H*)-furanone (2-FO), which is then hydrogenated separately to yield GBL. In addition, 2-FO is an important raw material for the production of surfactants, polymers, diols, and lactones^14–17^. Thus, many research groups have investigated the synthesis route of 2-FO. For example, Zvarych et al. achieved a 71% yield of 2-FO from FAL by using a 1:20 mixture of acetic acid and hydrogen peroxide (H_2_O_2_)^18^. Yu et al. developed a catalyst consisting of 20 wt% CuMoO_4_ and found that with a stoichiometric amount of peroxymonosulfate under 2 MPa air at 140 °C, the catalyst converted FAL into 2-FO with a yield of 66%^19^. However, although these methodologies have provided valuable mechanistic insights into the conversion of FAL or FA to GBL, they require stoichiometric oxidants, such as H_2_O_2_ and peroxymonosulfate, and harsh reaction conditions. This may reduce selectivity for 2-FO, leading to the production of various oxidised or ring-opening products, such as maleic acid (MA), 5-hydroxy-2(5H)-furanone (HFO), and CO_2_^20^. Thus, there is a need for a green and catalytic method of converting FAL or FA into GBL.

Recently, Wang et al. introduced a two-stage approach to convert FAL into GBL^6^. In the first stage, FAL was oxidised to 2-FO with excess H_2_O_2_ in an organic solvent at 60 °C. Next, 2-FO was isolated through vacuum distillation and subsequently hydrogenated to GBL using a 0.5 Pd/SiO_2_ catalyst under 3.5 MPa H_2_ at 80 °C, yielding 61.5% of 2-FO. During FAL oxidation, numerous byproducts were formed, such as maleic, succinic, lactic, and propionic acids, and over-reduction and ring-opening reactions afforded trace amounts of 2-hydroxytetrahydrofuran, methyl butyrate, and butanediol. This highlights that one-pot conversion of a furanic precursor to GBL via thermal catalysis can be challenging because it requires the redox reactions of various chemicals at different stages. As such, electrocatalysis involving specifically paired electrolysis is a promising solution because it uses an applied potential bias to effect simultaneous electrochemical redox reactions on each electrode. In recent years, paired electrolysis has attracted increasing interest due to its potential application in advanced electro-synthetic routes, and enhanced energy efficiency and economic value. Typically, most paired electrolysis setups involve separating redox reactions using an ion-exchange membrane or a porous frit to minimise yield loss from reverse reactions. The product streams are then collected separately or combined, depending on specific reaction requirements^21,22^. Separator-free one-pot paired electrolysis setups are relatively rare because they require careful substrate selection and precise condition tuning to suppress reverse reactions. Thus, only a few such setups have been reported and have used elaborate electrochemical mediators and had limited current flow, hindering the achievement of high yields and efficiency^23^.

In this study, we designed a one-pot paired electrolysis method to convert FA into GBL. We selected FA instead of FAL because the latter tends to be unstable in an acidic medium owing to its tendency to form humic substrates. In addition, under reductive conditions, FAL can easily be reduced to furfuryl alcohol^24^. However, FA does not exhibit these problems, and can be readily obtained from FAL via various mild methodologies. For instance, SiO_2_-Co(acac)_2_ catalysed the conversion of FAL into FA under solvent-free conditions, yielding an 85% isolated yield of FA within 5 h at 50 °C^25^. The solvent-free and heterogeneous nature of this method allowed the facile separation of FA. FA was also produced electrochemically from FAL in a 97% yield, with NiFe-1 as the anode and at 756 mA cm^−2^, a current density that is compatible with industrial-scale processes^26^. Biocatalytic methods can be employed; for example, *Pseudomonas putida* KT2440 converted FAL into FA in over 97% yield at pH 6 and 30 °C in over 2 h^27^. Additional examples of FAL-to-FA conversions are presented in Supplementary Table S3.

**Fig. 1:**
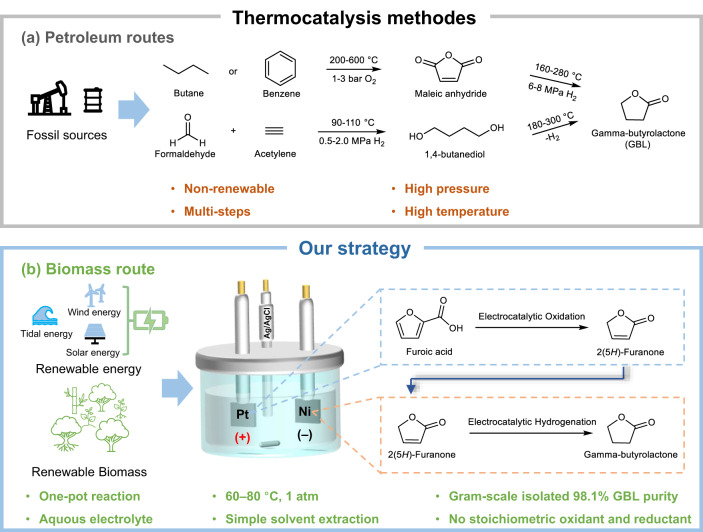
Reaction pathways to synthesis of gamma-butyrolactone (GBL). **a** The GBL synthesis from petroleum-derived substrates. **b** The production of GBL from biomass-relevant precursors proposed in this work.

In our approach, FA was first electrochemically oxidised to 2-FO, which was then electrochemically reduced in situ to GBL. We systematically investigated the influence of various reaction parameters on reactants and key intermediates to maximise the desired product selectivity from the respective electrodes and integrate redox reactions in an undivided cell. Through optimisation, we achieved a selectivity of 84.2% ± 2.4% for the conversion of FA to 2-FO and 98.5% ± 0.5% for the conversion of 2-FO to GBL. Both reactions were performed in an undivided cell under atmospheric conditions and at 80 °C using a platinum (Pt) anode paired with a nickel (Ni) cathode (Fig. 1). When both volume and concentration were increased, GBL could be isolated using a simple solvent extraction process. For example, in 500 mL of 100 mM FA, 2.1 ± 0.2 g of high-purity GBL was obtained (a 47.8% ± 3.5% isolated yield and a purity of 98.1% ± 0.4%). This mild one-pot paired electrosynthesis of GBL from FA is a green and sustainable approach for valorising furanic precursors, enabling their use in high-value applications^28^.

## Results and discussion

### Electrochemical oxidation of FA to 2-FO

The electrocatalytic conversion of FA to GBL involved the sequential oxidation of FA to 2-FO, followed by the reduction of 2-FO to GBL. A key factor that facilitated these two reactions in an undivided cell was that none of the involved species (e.g. FA, 2-FO, and GBL) underwent undesired redox reactions, due to the mildness of the conditions. Control experiments using cyclic voltammetry (CV) analysis and bulk electrolysis confirmed that FA could not be electrochemically reduced and could only be oxidised to 2-FO, which, in turn, could only be hydrogenated to GBL (Supplementary Fig. S1). This appropriate inertness of each chemical participant enabled the one-pot electrochemical transformation of FA to GBL; the corresponding redox chemical equations are provided in the SI.

**Fig. 2:**
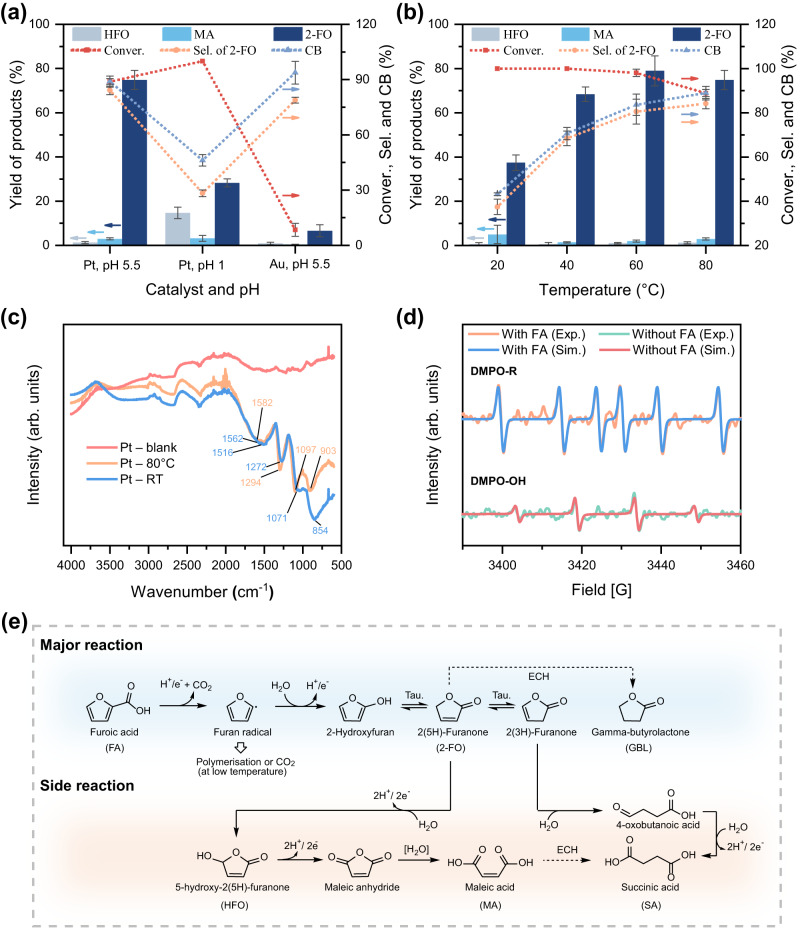
The ECO of FA under different conditions and the reaction mechanism. **a** ECO of FA at pH 1 or 5.5 on Pt or Au. **b** ECO of FA at temperatures ranging from 20 to 80 °C. Reaction conditions: 10 mM FA in 10 mL of pH 5.5 buffer; applied voltage: +1.8 V_Ag/AgCl_; Charge passed, 100 C; Pt foil as working and counter electrodes. Experiments were performed in triplicate and error bars correspond to the standard deviation of three independent measurements. **c** FTIR spectra at RT and 80 °C on Pt. **d** EPR spectra with and without FA, respectively, with Pt as the anode at 80 °C. **e** Proposed mechanistic pathway for the ECO of FA to GBL at 80 °C in pH 5.5 electrolyte.

**Fig. 3:**
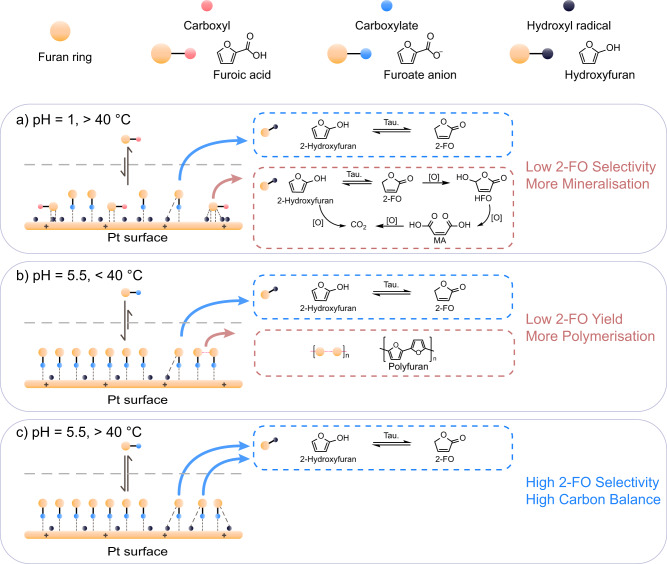
Various anodic interface events under different conditions. **a** At pH 1 and above 40 °C. **b** pH 5.5 and below 40 °C. **c** pH 5.5 and above 40 °C.

In preliminary experiments involving the time-resolved electrocatalytic oxidation (ECO) of FA at pH 2 using a Pt anode, we obtained yields of 83.6% ± 1.0% 2-FO, 11.6% ± 1.3% HFO, and 2.8% ± 0.4% MA (Supplementary Fig. S2). This distribution of products is consistent with that which has been reported in the literature^29–31^. All products were quantified through high-performance liquid chromatography (HPLC) with external calibration (Supplementary Fig. S3). A pH stability test revealed that 2-FO could undergo an irreversible ring-opening reaction to produce MA (Supplementary Fig. S4) at a pH greater than 6. Thus, the ECO of FA needed to be conducted in an acidic environment to maximise the preservation of 2-FO. At pH 1, the conversion of FA reached 100% but the 2-FO yield decreased to 28.3% ± 1.8% and the HFO yield increased to 14.7% ± 2.6% (from 11.6% ± 1.3% at pH 2) (Fig. 2a and Supplementary Table S1). The decreased yield of 2-FO may be attributable to weak furan ring-directed adsorption of FA, which promoted its overoxidation and mineralisation (Fig. 3a). This would have increased the HFO yield and the oxidative mineralising activity, as indicated by the low carbon balance (CB). To solve this problem, we used a pH of 5.5, at which FA was nearly entirely deprotonated, as indicated by an ultraviolet-visible spectroscopy analysis (Supplementary Fig. S5). At pH 5.5, the CB was 89.0% ± 1.6%, whereas at pH 1 it was only 46.1% ± 3.1% (Fig. 2a and Supplementary Table S1). The carboxylate form of FA, i.e., furoate anion, tended to adsorb in a bidentate manner and upright orientation onto the Pt surface (Fig. 3c)^29,32^, and promoted the ECO of the carboxyl group. The linear sweep voltammetry (LSV) analyses show a greater current density difference (with and without FA) at pH 5.5 than at pH 1, indicating a stronger furoate inhibition on Pt at pH 5.5, and thus confirming the enhancement of surface adsorption of the furoate anions at greater pH. (Supplementary Fig. S6).

Therefore, we selected pH 5.5 for the ECO of FA. A high pH can also suppress cathodic hydrogen evolution and promote the electrochemical hydrogenation (ECH) of organic compounds, which facilitates the ECH of 2-FO to GBL, as discussed later^33,34^. In addition, we found that using gold (Au) electrodes instead of Pt electrodes afforded a poor yield of 2-FO (6.5% ± 2.7%) (Fig. 2a and Supplementary Table S1). Density functional theory (DFT) calculations suggested that this was because the Au surface was more susceptible than the Pt surface to being ‘poisoned’ by upright furoate, thereby hindering furoate decarboxylation or oxidation steps^30^. In summary, our preliminary experiments indicated that the use of a Pt electrode at pH 5.5 was optimal for the ECO of FA to 2-FO.

The influence of temperature was examined by varying the temperature from 20 to 80 °C (Fig. 2b, Supplementary Table S2 for quantitative data) in experiments performed potentiometrically at a silver/silver chloride voltage (V_Ag/AgCl_) of +1.8 V  with the same coulomb of charge passed. At 20 °C, FA was completely consumed but only 37.5% ± 3.5% of 2-FO, 5.0% ± 4.2% of MA, and traces of HFO were formed, resulting in a poor CB. As the temperature increased from 20 to 40 °C, the yield of 2-FO increased to 68.4% ± 3.3%, the formation of MA decreased significantly, and the CB increased to 70.6% ± 2.8%. At 60 °C, the yield of 2-FO increased marginally to 79.0% ± 6.8%, as did the yields of MA and HFO, which increased the selectivity of 2-FO to 80.5% ± 5.6%. Overall, although the increase in temperature improved the yield of 2-FO, it decreased FA conversion, possibly because electrocatalytic oxidation shifted from FA oxidation to the oxygen evolution reaction (OER). To verify the increasing impact of OER on FA conversion at different temperatures, we conducted a series of LSV analyses at four designated temperatures in the absence of FA (Supplementary Fig. S7). Our results revealed that the anodic current increased with temperature. At +1.8 V_Ag/AgCl_, the current densities (*j*, mA cm^−2^) at 20, 40, 60, and 80 °C were 6.7, 11.9, 16.2, and 23.2 mA cm^−2^, respectively. The increase in current density with temperature is consistent with our understanding of the temperature-dependent activity of OER^35^.

At 80 °C, the most pronounced improvement in CB was observed, as it reached 89.0% ± 1.6%. The conversion of FA decreased slightly, from 98.0% ± 1.6% at 60 °C to 88.9% ± 3.0% at 80 °C. This reduction is attributable to the increasingly competitive nature of the OER. However, the yield of 2-FO decreased only slightly, to 74.8% ± 4.4%. Thus, both 2-FO selectivity and overall CB were highest at 80 °C.

In addition, we determined whether the decreased conversion of FA at elevated temperatures was due to the weakened adsorption of FA, as higher temperatures may facilitate its desorption^33,36^. Thus, we examined the change in the OER onset potentials in the presence and absence of FA, respectively, at designated temperatures through LSV. At *j* = 7.5 mA cm^−2^, the OER onset potential differences in the presence and absence of FA, respectively, at 20, 40, 60, and 80 °C were +168, +156, +120, and +77 mV, respectively (Supplementary Fig. S8). Under a positive potential bias, the furoate anion can form an inhibitory film on the positively charged anode, thereby shifting the OER onset potential to a more positive value than before^37^. However, as the temperature increased, the onset potential shift diminished, indicating that the inhibition of OER by the FA film was weakened. Thus, we concluded that the lower FA conversion at higher temperatures resulted from both enhanced competition with the OER and weakened FA adsorption.

Although elevated temperatures could reduce the conversion of FA, they still played a crucial role in promoting 2-FO production and improving the CB. At 20 °C, although FA was completely converted, only a small fraction became 2-FO. This indicated that FA underwent polymerisation on the Pt anode instead of being oxidised to 2-FO. To confirm the deposition of polymers, we employed ex-situ attenuated total reflectance Fourier-transform infrared spectroscopy (ATR-FTIR) to examine the post-electrolysis Pt surfaces from trials conducted at 20 and 80 °C, respectively (Fig. 2c). No carbonyl (C=O peak) (~1710 cm^−1^) was observed, indicating that no carbonyl-bearing compounds, such as 2-FO, HFO, FA, and MA, were adsorbed onto the surface. However, the presence of peaks at 1582 and 1516 cm^−1^ indicated the presence of furan olefinic (C=C) bonds^38,39^. In addition, peaks at 1562, 1294, 1272, 1097, and 1071 cm^−1^ represented the furan carbon–oxygen bond in the ring^38–42^. The region from 760 to 1025 cm^−1^ indicated the presence of disubstituted furan^43^, which resulted from the polymerisation of furan. Furthermore, the greater number of peaks in the region from 760 to 1025 cm^−1^ observed in trials conducted at 20 °C compared with those conducted at 80 °C confirmed that there was more coverage of polyfuran at 20 °C than at 80 °C. This observation is consistent with the poor CB observed at low temperatures.

A general mechanistic model of FA oxidation in an aqueous environment showed that the formation of 2-FO begins with the electrocatalytic decarboxylation of FA, resulting in the formation of a furan radical intermediate. This intermediate can either be transformed into 2-hydroxyfuran, which tautomerises to 2-FO, or undergo anodic polymerisation or mineralisation if reaction temperature is low, thereby reducing the CB^19,44^. (Fig. 2e) This model led us to hypothesise that the yield of 2-FO depends on the fate of the furan radical intermediate. We first verified the formation of the furan radical in our ECO system through electron paramagnetic resonance (EPR) spectroscopy. The results revealed the formation of a carbon-centred radical adduct 5,5-dimethyl-1-pyrroline *N*-oxide (DMPO-R; A_N_ = 15.3 G and A_H_ = 24.7 G) in the presence of FA (Fig. 2d)^45,46^, providing strong evidence for the presence of the furanic radical. However, in the absence of FA, EPR showed only the DMPO-OH adduct (A_N_ = 14.9 G and A_H_ = 14.9 G) under the same reaction conditions. These results suggest that after decarboxylation, the furan radical combines with the OH radical on the Pt surface to yield 2-hydroxyfuran, which subsequently undergoes tautomerisation to form 2-FO.

At 20 °C, FA was completely consumed but it mainly polymerised into polyfuran, resulting in a low CB. Changing the temperature from 20 to 40 °C significantly improved the CB because the yield of 2-FO almost doubled, from 37.5% ± 3.5% to 68.4% ± 3.3%. However, a further increase in temperature to 60 and 80 °C only led to a minor increase in the 2-FO yield, to 79.0% ± 6.8% and 74.8% ± 4.4%, respectively. The marked improvement in the yield of 2-FO at 40 °C but not 60 and 80 °C is attributable to the fact that the boiling point of furan is 31.3 °C. That is, at 20 °C, the furan radical remained on the electrode surface, which led to its polymerisation (Fig. 3b). However, at temperatures greater than 40 °C, the volatility of the furan radical dominated, resulting in enhanced formation of 2-hydroxyfuran and 2-FO (Fig. 3c). At 60 and 80 °C, the yields of 2-FO were similar because both temperatures exceeded the boiling point of furan and its radical. A similar temperature-dependent observation was made in a study on the (electro)chemical oxidative formation of polyfuran in organic solvents^47^. It was found that polyfuran formation was increased as temperature increased up to the boiling point of furan, but that the yields of polyfuran and oligofuran substantially decreased at temperatures above 32 °C^48^. In an aqueous electrolyte system at elevated temperatures, the volatile furan radical presumably escapes from the electrode surface and reacts with the surrounding H_2_O to yield 2-hydroxyfuran and 2-FO. Thus, an increase in temperature prevented the furan radical intermediate from polymerising, leading to 2-FO formation.

In summary, we demonstrated the electrocatalytic oxidation of FA to 2-FO. Our findings suggest that the reaction should be catalysed by Pt at pH 5.5 and 80 °C to achieve a balance between a good yield (74.8% ± 4.4%), selectivity for 2-FO (84.2% ± 2.4%), and a good CB (89.0% ± 1.6%). Alkaline pHs should be avoided, due to the instability of 2-FO at pHs higher than 6. Similarly, strongly acidic electrolytes (e.g. pH 1) should be avoided because they would lead to the overoxidation of FA. At pH 5.5, the carboxylate group adsorbs perpendicularly onto the surface in a bidentate fashion and thus undergoes decarboxylation (-CO_2_) via a radical rearrangement upon oxidation. This process yields a furan radical, which then reacts with H_2_O from the bulk electrolyte to form hydroxyfuran. Subsequently, hydroxyfuran tautomerises to form 2-FO. Increasing the temperature beyond 40 °C promotes the thermal desorption of the furan radical, enhancing the selectivity towards 2-FO.

### Electrocatalytic hydrogenation of 2-FO to GBL

**Fig. 4:**
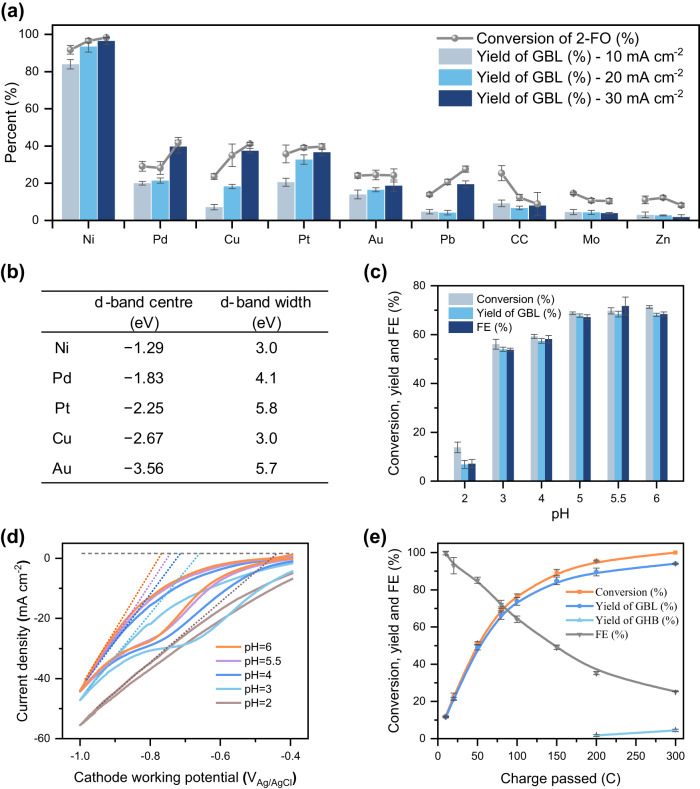
ECH of 2-FO under different conditions. **a** ECH of 20 mM 2-FO in 20 mL of 0.5 M buffer (pH 5.5) at 80 °C on a Ni cathode paired with a Pt anode as a counter electrode with electrolysis at 10, 20 and 30 mA cm^−2^ with a total of 360 C passed. **b** The d-band centre and width of bulk electrocatalysts. **c** conversion and yield at pH 2–6 (2.0 V_Ag/AgCl_; WE: Pt, CE: Ni) with a total of 100 C passed. **d** CV analysis of 20 mM 2-FO at 80 °C at a scan rate of 50 mV s^−1^ (WE: Ni, RE: Ag/AgCl, CE: Pt). **e** Time-resolved electrolysis of 2-FO (2.0 V_Ag/AgCl_; WE: Pt, CE: Ni) under the optimised conditions (pH 5.5 buffer at 80 °C). Experiments were performed in triplicate and error bars correspond to the standard deviation of three independent measurements.

The electrocatalytic oxidation of FA to 2-FO was selective at an elevated temperature. However, the Pt cathode could not reduce 2-FO efficiently because it preferentially facilitated the HER. Thus, we conducted a search for a cathode material capable of selectively hydrogenating the olefin of 2-FO instead of its carbonyl group (Fig. 4a). We investigated the performance of eight common ECH metals and carbon cloth (CC) as cathodes at current densities of 10, 20, and 30 mA cm^−2^, respectively, which corresponded to the estimated current generated between the anodic potentials of +1.7 to +2.0 V_Ag/AgCl_. In addition, 360 C (4.6 times the reducing equivalence) was passed to maximise reaction completion. The 2-FO conversion and GBL yield were measured to examine the relative performance of the various electrocatalysts. As illustrated in Fig. 4a, Ni was the most efficient catalyst for the ECH of the olefin in 2-FO, affording 84.0% ± 2.5%, 93.5% ± 3.0%, and 96.5% ± 1.9% of GBL at 10, 20, and 30 mA cm^−2^, respectively. The GBL yield matched the 2-FO conversion at 30 mA cm^−2^, indicating that GBL was produced with a considerably high selectivity, i.e., 98.2% ± 1.8%. This is attributable to the favourable absorption of C=C on its surface because desorption of C=O is more facile than desorption of C=C^49^. The high C=C ECH efficiency can be explained by the d-band model devised by Nørskov et al.^50^. According to this model, a short distance between a d-band centre and a Fermi level enhances the binding energy between a metal surface and absorbents (Fig. 4b). Among the top five most active metal catalysts, the binding energy of 2-FO followed the trend of Ni <Pd <Pt <Cu <Au, which closely aligned with the GBL yields observed in the experiment (Fig. 4a). Delbecq et al.^51^ reported that a decrease in the d-band width increased the interaction between the C=C bond and the metal surface, resulting in increased selectivity for C=C hydrogenation over C=O reduction. Among the five metal catalysts, Ni possessed the narrowest d-band, consistent with our experimental observation for C=C hydrogenation. To verify the electrocatalytic reduction activity for 2-FO hydrogenation, we performed CV analysis of all of the cathode materials (Supplementary Fig. S9). Only the CVs of Ni displayed an observable difference in current with and without 2-FO, respectively, aligning with the aforementioned d-band width model of the C=C absorption of 2-FO onto Ni.

We next examined the influence of temperature from 20 to 80 °C (Supplementary Fig. S10). As the temperature increased from 20 to 80 °C, the conversion of 2-FO increased from 12.8% ± 0.8% to 69.8% ± 1.3% and the yield of GBL reached 68.4% ± 1.2%. These results indicate that an increase in temperature promoted the surface desorption of 2-FO^34^, allowing the surface to regenerate the adsorbed H_2_ needed for C=C hydrogenation. The high GBL yield observed at 80 °C was ideal for coupling the ECO of FA with the ECH of 2-FO because both reactions were favoured by high temperatures.

We investigated the influence of pH on 2-FO reduction from pH 2 to 6 at 80 °C (Fig. 4c). Neutral and alkaline conditions were not considered because 2-FO is chemically unstable beyond pH 6, as found in an earlier control experiment (Supplementary Fig. S4). As the pH decreased, the yield of GBL decreased, from 68.1% ± 0.7% at pH 6 to 57.4% ± 1.0% at pH 4 and to 6.8% ± 1.6% at pH 2. This reduction in GBL yield is attributable to the increased coverage of surface adsorbed hydrogen (H_ads_) on the Ni cathode in an acidic environment, which shifted selectivity from the ECH of 2-FO to the HER. A series of CV experiments were conducted to observe changes in onset HER and 2-FO ECH potentials (Fig. 4d). At pH 2, the ECH of 2-FO was inefficient, and thus CV analysis only had HER curves starting at approximately −0.44 V_Ag/AgCl_. However, starting from pH 3, where the ECH of 2-FO became efficient, a distinct peak reflecting the electrochemical reduction of 2-FO appeared at −0.65 V_Ag/AgCl_. This peak was not observed in the absence of 2-FO (Supplementary Fig. S11). As the pH increased, the HER onset potential and the 2-FO reduction peaks became increasingly negative until pH 5.5. The onset potential for the HER became increasingly more negative than that for 2-FO, indicating minimal competition from the HER. This explains why the Faradic efficiency (FE) of the ECH of 2-FO improved as the pH increased (Fig. 4c).

To better assess the compatibility between the ECO of FA and the ECH of 2-FO, we examined the reduction efficiency of 2-FO in relation to the Pt anodic working potential (Supplementary Fig. S12). The results demonstrated that the ECH of 2-FO tolerated a wide range of anodic potentials, ranging from +1.8 to +2.0 V_Ag/AgCl_, which is highly advantageous for the successful coupling of the ECO and ECH reactions.

A time-resolved electrolysis was conducted under optimised conditions to investigate the change in product distribution and FE for 2-FO reduction (Fig. 4e). From 0 to 150 C, all the 2-FO was converted into GBL, with no side products formed, as indicated by the overlapping curves of the 2-FO conversion and GBL yield. However, beyond 150 C, the two curves began to diverge, suggesting that GBL was lost due to hydrolysis. At the end of electrolysis, all of the 2-FO was consumed and hydrogenated at the olefin site with a selectivity of 98.5% ± 0.5%. This resulted in the production of 94.1% ± 0.3% GBL and 4.4% ± 0.7% GHB. GHB was formed through the ring-opening hydrolysis of GBL, which is a side reaction that commonly occurs at pHs greater than 2^52^. Trace amounts of overly oxidised products, such as HFO and MA, were also detected (Supplementary Fig. S13). The FE was calculated based on detected products, and it gradually declined as 2-FO diminished, indicating a shift in selectivity from the ECH of 2-FO to the HER.

In summary, the ECH of 2-FO demonstrated high efficiency across a range of working potentials, temperatures, and pHs. Using a Ni cathode, there was high selectivity for ECH of the C=C bond in 2-FO (98.5% ± 0.5%) with complete conversion. Time-resolved electrolysis showed that the selectivity for 2-FO C=C hydrogenation was maintained throughout electrolysis, but the FE gradually declined with the depletion of 2-FO. The ECH of C=C is highly compatible with the ECO of FA, enabling the one-pot conversion of FA to GBL.

### Integration of FA Oxidation and 2-FO Reduction for Direct GBL Production

**Fig. 5:**
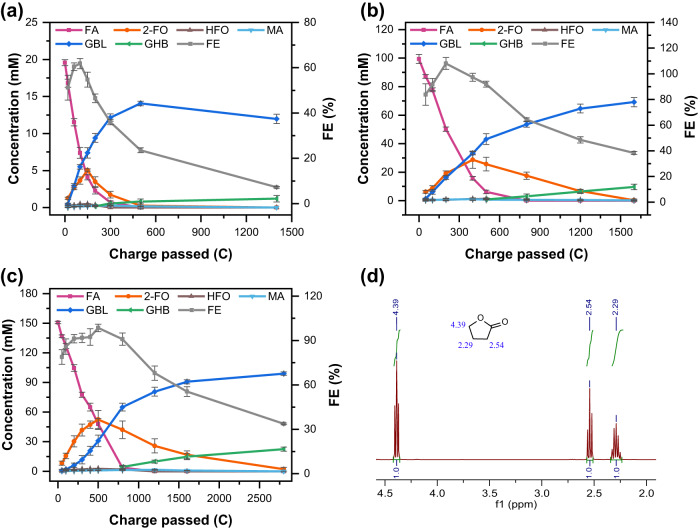
One-pot electrochemical conversion of FA with different charges passed. **a** 20 mM FA, **b** 100 mM FA, **c** 150 mM FA in 20 mL (pH 5.5) buffer; 80 °C; 2.0 V _Ag/AgCl_; WE: Pt, CE: Ni. Experiments were performed in triplicate and error bars correspond to the standard deviation of three independent measurements. **d** ^1^H-NMR analysis of isolated GBL in CDCl_3_ (full spectrum given in Supplementary Fig. S15); reaction conditions: 50 mA cm^−2^, 24 h, 3 ×3-cm Pt anode paired with 3 ×3-cm Ni cathode, pH 5.5, 80 °C.

**Fig. 6:**
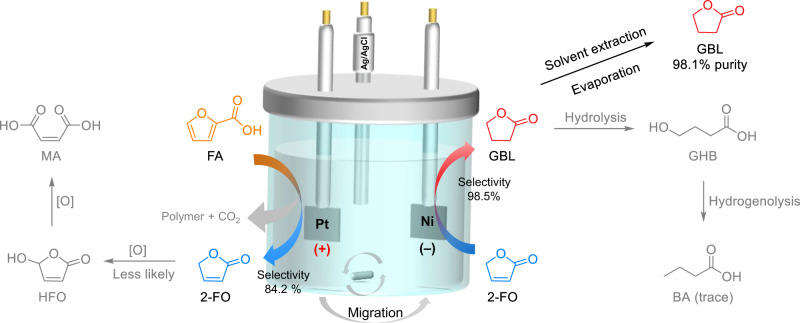
Electrochemical redox cascaded synthesis of GBL in one-pot. From furoic acid (FA) to 2(5*H*)-furanone (2-FO) to gamma-butyrolactone (GBL).

The one-pot electrochemical transformation of FA to GBL was performed under optimised conditions of 80 °C and +2.0 V_Ag/AgCl_ with the Pt anode (Fig. 5). Three initial FA concentrations (20, 100, and 150 mM) were examined. As expected, all trials showed that FA was oxidised to 2-FO on the Pt anode. Then, 2-FO was reduced to GBL on the Ni cathode (Fig. 6). When the initial concentration of FA was 20 mM, the 2-FO yield peaked at 25.9% ± 0.5% (5.1 ± 0.1 mM) after 150 C was passed, and then gradually decreased as GBL production increased at the expense of 2-FO (Fig. 5a). The GBL yield reached 71.9% ± 2.5% (14.1 ± 0.3 mM) after 500 C was passed and then declined progressively due to the hydrolytic ring-opening reactions over an extended period. When an initial FA concentration of 100 mM was used (Fig. 5b and Supplementary Fig. S14), 2-FO grew rapidly initially and then was hydrogenated to GBL. The GBL yield reached 69.1% ± 3.2% with 38.3% ± 1.0% FE and an 80.1% ± 1.8% CB after 1600 C were passed. When the initial FA concentration was 150 mM, the GBL yield reached 98.9 ± 1.7 mM (Fig. 5c). The trial involving the use of 150 mM FA resulted in a dark yellow electrolyte, indicating the occurrence of furanic polymerisation, and no products were detectable via gas chromatography–mass spectrometry^19^. Thus, the initial concentration of FA should be limited to 100 mM.

A scaled-up volume of 500 mL of 100 mM FA (5.6 g in 500 mL) was used under optimised conditions to generate sufficient GBL for product isolation, with 38,880 C delivered to maximise the conversion of FA to GBL. After dichloromethane (DCM) extraction and subsequent DCM removal in vacuo, 2.1 ± 0.2 g (47.8% ± 3.5% isolated yield) of GBL with a purity of 98.1% ± 0.4% was obtained (as determined by ^1^H-NMR; partial spectrum, Fig. 5d; full spectrum, Supplementary Fig. S15). This sub-optimal isolated yield likely resulted from the hydrolysis of GHB due to prolonged electrolysis and loss of GBL during removal of DCM under vacuum. GHB and succinic acid (SA) were also detected near the end of the reaction as FA and 2-FO became depleted, and based on pre-extracted ^1^H-NMR analysis, the GHB and SA yields were 12% and 5%, respectively (Supplementary Fig. S16). SA might be generated from the ring-opening of 2(3*H*)-furanone, which yields 4-oxobutanoic acid, followed by the ECO of its aldehyde group^32^. It may also be formed from the ECH of the C=C bond of MA, which came from HFO described in Fig. 2e. A trace of butyric acid (BA) was also observed, which might be generated from the reduction of GHB. DCM extraction was highly selective for GBL^53^, as demonstrated by the high purity mentioned above. Efforts to reduce the electrolysis time required for high-volume synthesis are currently underway. Nevertheless, our one-pot FA-to-GBL method generated highly pure GBL at a rate compatible with those of existing FAL-to-FA technologies. Based on the conversion rate, our scaled-up reaction consumed 5.6 g of FA per 24 h, which is equivalent to 2083.3 µmol/h. A thermal catalytic method using MnO_2_@CeO_2_ under 8 bar O_2_ at 130 °C converted FAL to FA at a rate of 750 µmol/h^54^. Biocatalytic methods could also provide high FAL to FA conversion rates, such as 2425 µmol/h^27^. Our one-pot FA-to-GBL electrosynthesis shows good compatibility in reaction rates with some of the existing FAL-to-FA technologies, which will allow their integrations with our synthesis of GBL.

In conclusion, an one-pot electrocatalytic method was developed to efficiently transform FA directly into GBL, a high-value compound. FA was first electrochemically oxidised to produce 2-FO, which was then hydrogenated to yield GBL in the same electrolyte. The reaction occurred in an undivided cell at 60–80 °C under atmospheric pressure and did not require the addition of chemical redox reagents. An elevated temperature beyond the boiling point of furan was essential for the effective production of 2-FO because such temperatures increased the volatility of the furan radical intermediate, facilitating its reaction with H_2_O from the bulk environment to yield 2-FO. The impact of reaction conditions, namely cathode material, reaction temperature, electrolyte pH, and applied potentials, was evaluated and systematically optimised. FA was electrochemically oxidised with a selectivity of 84.2% ± 2.4% to 2-FO, which was then in-situ hydrogenated at the olefin site to yield GBL with a selectivity of 98.5% ± 0.5%. During extended electrolysis, GBL was susceptible to hydrolysis, leading to the formation of ring-opened products that decreased the CB of the reaction. Thus, the initial FA concentration should be limited to 100 mM to reduce reaction time. At 20-mL benchtop scale, the highest yields of GBL were 71.9% ± 2.5% and 69.1% ± 3.2% at 20 mM and 100 mM, respectively. In a scaled-up synthesis comprising 5.6 g of FA in a 500-mL solution, 2.1 ± 0.2 g of GBL with a purity of 98.1% ± 0.4% was obtained. The one-pot and facile transformation of FA to GBL represents a green and scalable method for valorising biomass-derived feedstock, illustrating the key role of biorefineries in the production of valuable platform chemicals.

## Methods

### Materials

All solutions were prepared using ultrapure deionised water (>18.2 MΩ cm^−1^, Millipore). Potassium hydrogen phosphate (K_2_HPO_4_, 99%), potassium phosphate monobasic (KH_2_PO_4_, 99.8%), 2(5H)-furanone (2-FO, 98%), and maleic acid (MA, >99%) were purchased from Aladdin. Phosphoric acid (85%–87%), methanol (ACS grade), and dichloromethane (DCM, ACS grade) were purchased from Anaqua. Butyric acid (BA, 99%) and furoic acid (FA, 98%) were purchased from Dieckmann. Formic acid (*>*99%) and 5-hydroxy-2(5H)-furanone (HFO, 98%) were purchased from Macklin. All reagents and the electrodes purchased from commercial sources were used without additional purification or modification.

### Electrocatalytic reaction

An electrochemical workstation (CHI 660E, Shanghai CH Instruments Co., China) was employed. All electrolysis experiments were performed in a 30-mL undivided cell with a three-electrode configuration. All metal electrodes were cleaned in acetone and in water for 5 min in ultra-sonication condition, followed by immersing them in 0.5 M H_2_SO_4_ for 2 min. All potentials reported in this work were referenced to an Ag/AgCl reference electrode without iR-compensation. Electrolyte solutions with different pHs were prepared by mixing 0.5 M H_3_PO_4_, KH_2_PO_4_, and K_2_HPO_4_. Unless otherwise specified, each electrolysis experiment employed an anode and a cathode with dimension of 10 ×10 ×0.1 mm with both sides uncovered.

### Scaled-up electrolysis and product isolation

The scaled-up reaction was conducted in a 1000-mL single cell with two electrodes (3 ×3-cm Ni cathode; 3 ×3-cm Pt anode) operating galvanostatically at 50 mA cm^−2^. The same electrolyte as mentioned above was used (pH 5.5), except that 5.6 g of FA was used in a 500-mL electrolyte (100 mM). After 24 h of electrolysis, the reaction mixture was extracted with 1000 mL of DCM, the organic layer was dried over anhydrous sodium sulphate, and then DCM was removed by rotary evaporation for 1 h to obtain GBL (2.1 g, 47.8% yield).

### Product analysis

Quantitative analyses of all products were conducted using a Waters Breeze HPLC instrument. A reverse-phase column (C18, Atlantis) operated at 30 °C was used to separate the product mixture. The mobile phase was a 10 mM aqueous solution of H_3_PO_4_/KH_2_PO_4_ and methanol in a 90:10 (v/v) ratio at an isocratic flow of 1 mL/min. The concentrations of FA and its products were quantified with external standards using a photodiode array and refractive index detectors. Infrared spectra were collected using a Fourier-transform infrared spectrometer (Perkin Elmer), with four scans performed at 4500–600 cm^−1^. UV–vis spectrophotometry was performed using a UV-3600 spectrophotometer (Shimadzu, Japan) in the 220–300-nm range. The purity of GBL was determined by ^1^H-NMR spectroscopy (Bruker Advance-III) using a 400-MHz instrument equipped with a broadband probe and referenced to a formic acid external standard.

### Calculations

The conversion (Conv.), selectivity (Sel.), CB, FE, and yield were calculated using the following equations:

1 $${Conv}.\left(\%\right)=\left(1-\frac{{{mol}}_{{reactant}}}{{{mol}}_{{reactant} \, {init}.}}\right)\times 100\%$$

2 $${CB}\left(\%\right)=\frac{\sum {{mol}}_{{products}}\,}{{{mol}}_{{reactant}\,{init}.}}\times 100\%$$

3 $${{Sel}.}_{x}\left(\%\right)=\frac{{{mol}}_{x}}{{{mol}}_{{reactant}\,{init}.}-{{mol}}_{{reactant}}\,}\times 100\%$$

4 $${FE}\left(\%\right)=\frac{n\times F\times \sum {{mol}}_{{products}}\,}{\iint {dIdt}}\times 100\%$$

5 $${{Yield}}_{x}\left(\%\right)=\frac{{{mol}}_{x}}{{{mol}}_{{reactant}\,{init}.}}\times 100\%$$

Where mol_*reactant init*_. and mol _reactant_ is the moles of the corresponding reactant before and after the reactions, respectively; mol_x_ is related to the moles of products; n and F are the electron transfer number and the Faraday constant at 96,485 C/mol, respectively. The total charge was calculated from the integral of the current (I, A) with respect to the operation time in seconds.

### Supplementary information

Supplementary Information

Peer Review File

### Source data

Source Data

## Data Availability

All data supporting the findings of this study are available within the main text and the Supplementary Information, and source data are provided with this paper. These data are also available from the authors upon request. Source data are provided with this paper.
