# Supplementary material for: One-pot redox cascade paired electrosynthesis of gamma-butyrolactone from furoic acid
Source: Nat Commun. 2024 Feb 7;15:1141. doi: 10.1038/s41467-024-45278-z (PMC10850494; doi:10.1038/s41467-024-45278-z)
Supplement: Supplementary file 1 — Supplementary Information [file 41467_2024_45278_MOESM1_ESM.pdf]

Supporting Information

One-Pot Redox Cascade Paired Electrosynthesis of Gamma-Butyrolactone from Furoic Acid

Shengqin Liu, [1] **Yangxin Jin,** [1] **Shuquan Huang,** [1, 2] **Qi Zhu,** [1] **Shan Shao,** [1] **and Jason Chun-Ho Lam***[1, 3]

[1] School of Energy and Environment, City University of Hong Kong, Kowloon Tong, Hong Kong SAR 999077, China

[2] Faculty of Chemical Engineering, Kunming University of Science and Technology, Kunming 650500, China

[3] State Key Laboratory of Marine Pollution, City University of Hong Kong, Kowloon Tong, Hong Kong SAR 999077, China

***Corresponding author:** jason.lam@cityu.edu.hk

Content

Redox chemical equations 1

Table S1. Electrocatalytic oxidation of FA at different anode and pH 2

Table S2. Electrocatalytic oxidation of FA at different temperatures........ 2

Table S3. Comparison of different systems for oxidation of furfural to produce furoic acid... 2

Fig. S1. Cyclic voltammograms (CVs) on Pt and Ni 3

Fig. S2. The electrochemical oxidation of FA in pH 2........ 4

Fig. S3. HPLC calibration curves of different products 5

Fig. S4. pH stability examination of 2-FO in pH 2 to 9 6

Fig. S5. (a) The UV-Vis absorbance of FA (0.1 mM) **(b)** The maximum UV-Vis absorbance (λ max) of FA from pH 2 to 9. 6

Fig. S6. LSV study at pH 1 and pH 5.5 with or without 50 mM FA........ 7

Fig. S7. Linear sweep voltammograms (LSVs) at 20 to 80 °C temperature range. 7

Fig. S8. Linear sweep voltammograms (LSVs) curve of the electrode in pH 5.5 buffer with and without 50 mM of FA at 20 to 80 °C temperature range. 8

Fig. S9. Cyclic voltammograms (CVs) in the presence and absence of 20 mM of 2-FO in 20 mL pH 5.5 electrolyte at 80 °C using different working electrodes 9

Fig. S10. The electrochemical reduction (ECH) of 2-FO at 20 – 80 °C........ 10

Fig. S11. Cyclic voltammograms (CVs): With and without 20 mM 2-FO in pH 5.5 electrolyte at 80 °C.. 10

Fig. S12. The electrochemical reduction (ECH) of 2-FO at an applied potential of 1.6 to 2.0 $V_{Ag/AgCl}$ 11

Fig. S13. HPLC chromatograms of PDA @ 210 nm sample analyses........ 12

Fig. S14. HPLC chromatograms of (a) -PDA @ 210 nm and (b) -RI sample analyses 12

Fig. S15. The purity of GBL was examined by ^{1}H NMR........ 13

Fig. S16. ^{1}H NMR analysis of pre-extracted electrolyte in D_2O. 14

Supplementary Reference 15

Redox chemical equations

During the reduction of 2-FO (Eq. 1), the hydrogen evolution reaction (Eq. 2) occurred in parallel on the cathode as well. As for the anodic processes, water splitting (Eq. 3) was the sole reaction since 2-FO could not be oxidized as control experiments verified. The electrochemical equations are as follows:

Cathode

2-FO + $2H^+$ + $2e^-$ --> GBL   Eq. 1

$2H^+$ + $2e^-$ --> H_2 Eq. 2

Anode

H_2O --> O_2 + $4H^+$ + $4e^-$ Eq. 3

During the FA oxidation study in unpaired mode, furan-2-carboxylate anion (FA anion) underwent a single-electron oxidation to induce decarboxylation (Eq. 4) to become a furan radical, which reacts with water to yield 2-FO. (Eq. 5) The cathodic reaction would be a hydrogen evolution reaction. (Eq. 2) The electrochemical equations are as follows:

Anode

Furan-2-carboxylate ---> Furan radical + CO_2 + e^- Eq. 4

Furan radical + H_2O --> 2-FO + H^+ + e^- Eq. 5

Cathode

$2H^+$ + $2e^-$ --> H_2 Eq. 2

Table S1. Electrocatalytic oxidation of FA at different anode and pH

Entry	WE	pH	Conversion (%)	Yield of products (%)			Sel of 2-FO (%)	CB (%)
				HFO	MA	2-FO		
1	Pt	1	100 ± 0.0	14.7 ± 2.6	3.2 ± 1.3	28.3 ± 1.8	28.3 ± 1.8	46.1 ± 3.1
2	Pt	5.5	88.9 ± 3.0	1.3 ± 0.5	3.0 ± 0.5	74.8 ± 4.4	84.2 ± 2.4	89.0 ± 1.6
3	Au	5.5	8.4 ± 3.6	0.8 ± 0.6	0.4 ± 0.2	6.5 ± 2.7	77.3 ± 4.0	92.2 ± 4.1

Reaction conditions: 10 mM FA in 10 mL of 0.5 M buffer at +1.8 $V_{Ag/AgCl}$ referenced to working anode (WE) paired with a Pt counter electrode at 80 °C.

Table S2. Electrocatalytic oxidation of FA at different temperatures

Entry	Tem. (°C)	Conver. (%)	Yield of products (%)			Sel. (%)	CB (%)
			HFO	MA	2-FO		
1	20	100 ± 0.0	0.7 ± 0.6	5.0 ± 4.2	37.5 ± 3.5	37.5 ± 3.5	43.2 ± 0.7
2	40	100 ± 0.0	0.7 ± 0.6	1.4 ± 0.3	68.4 ± 3.3	68.4 ± 3.3	70.6 ± 2.8
3	60	98.0 ± 1.6	1.1 ± 0.3	2.0 ± 0.5	79.0 ± 6.8	80.5 ± 5.6	83.7 ± 4.9
4	80	88.9 ± 3.0	1.3 ± 0.5	3.0 ± 0.5	74.8 ± 4.4	84.2 ± 2.4	89.0 ± 1.6

Reaction conditions: 10 mM FA in 10 mL of 0.5 M pH 5.5 buffer at +1.8 $V_{Ag/AgCl}$ referenced to the Pt working anode paired with a Pt counter electrode at 80 ºC.

Table S3. Comparison of different systems for oxidation of furfural to produce furoic acid.

Entry	Catalyst	Reaction conditions	Performance	Ref.
1	SiO_2-Co(acac)$_2$	Solvent-free, 50 °C, air, 5 h	85% isolated FA yield, 170 μmol/h	1
2	BTIC	125 mM FAL, DMSO with DBU, 40 °C, 0.1 MPa O_2, 4 h	> 99% FA yield, 62.5 μmol/h	2
3	MnO_2@CeO_2	300 mM FAL, H_2O, 130 °C, 8 bar O_2, 4 h	96% FA yield, 750 μmol/h	3
4	$Fe^{III}Mo_6$	750 mM FAL, 0.75 M $NaCO_3$, 50 °C, 1 atm O_2, 8 h	97% isolated FA yield, 182 μmol/h	4
5	NiFe-1	100 mM FAL, 1.478 V vs. RHE, 1 M KOH	97% FA yield, 96% FE, 756 mA cm^{-2}	5
6	NiMoP/NF	100 mM FAL, 1.46 V vs. RHE, 1 M NaOH	99.2% FA yield, 96.7% FE, 500 mA cm^{-2}	6
7	Ni_xSe_y–NiFe LDH@NF	20 mM FAL, 1.423 V vs. RHE, 1 M KOH	99.7% FA yield, 99.5% FE, ~ 100 mA cm^{-2}	7
8	Ag_2O@Ni foam	530 mM FAL, 1.95 V OCV, 1 M KOH	90% FA yield, 94% FE, 400 mA cm^{-2}	8
9	*Comamonas testosteroni* SC1588	50 mM FAL, 30 °C, pH 7 buffer, 10 h	96% FA yield, 20 μmol/h	9
10	*P. putida* KT2440	204 mM FAL, 30 °C, pH 6 buffer, 3 h	>97% FA yield, 2425 μmol/h	10

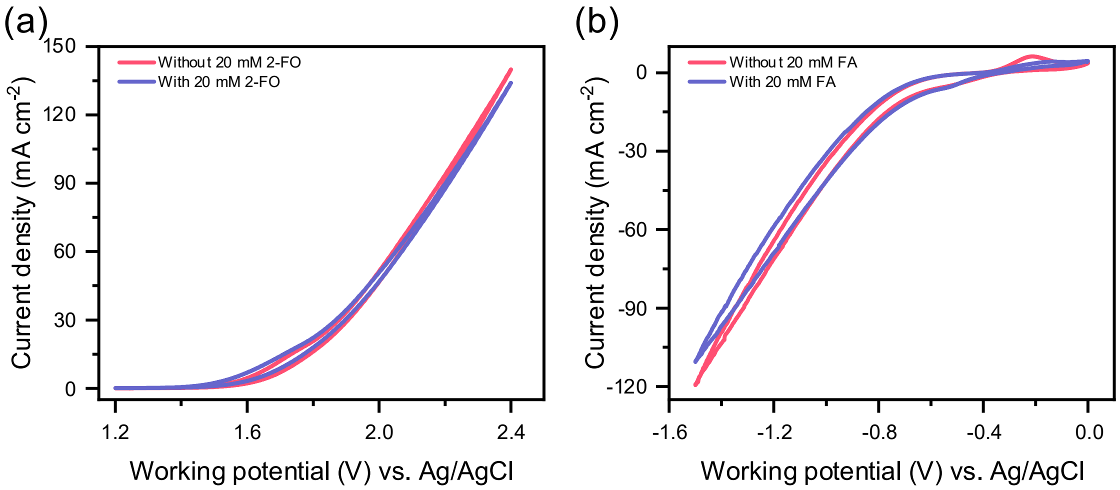

Fig. S1. Cyclic voltammograms (CVs) on Pt and Ni: **(a)** Positive scan using Pt as working electrode with and without 20 mM 2-FO and **(b)** Negative scan using Ni as working electrode with and without 20 mM FA in 20 mL pH 5.5 electrolyte at 80 °C respectively.

Preliminary Experiment: Product Identification

The time-resolved electrochemical oxidation (ECO) of 2-furoic acid (FA) galvanometric electrolysis was conducted using the Pt anode paired with a Pt cathode operating in pH 2 at 1 mA to investigate the change in Faradaic Efficiency (FE) and product distribution (Fig. S2). The Pt cathode was selected because the control experiment revealed it performed H^+ reduction to H_2, aka. hydrogen evolution reaction (HER), almost exclusively instead of 2-FO in pH 2, which allowed us to analyze the 2-FO product more accurately. As the passed charge increased from 0 to 43.2 C (2.24 equivalence oxidizing charge), the FA conversion reached 89.6 ± 0.8 % and 73.7 ± 2.0 % 2-FO was produced. The reaction also produced 12.1 ± 1.1 % HFO and 2.6 ± 0.2 % MA. As the charge delivery increased to 86.4 C (4.5 oxidizing equivalent charge), all FA was consumed and yielded 83.6 ± 1.0 % of 2-FO, accompanied by a small amount of HFO and MA. This result suggested the side-reaction includes the over-oxidation of 2-FO to HFO and MA in this low current density (1 mA cm^{-2}) and acidic pH. The FE decreased as the FA diminished, which shifted the electrochemical reaction from oxidizing FA to water.

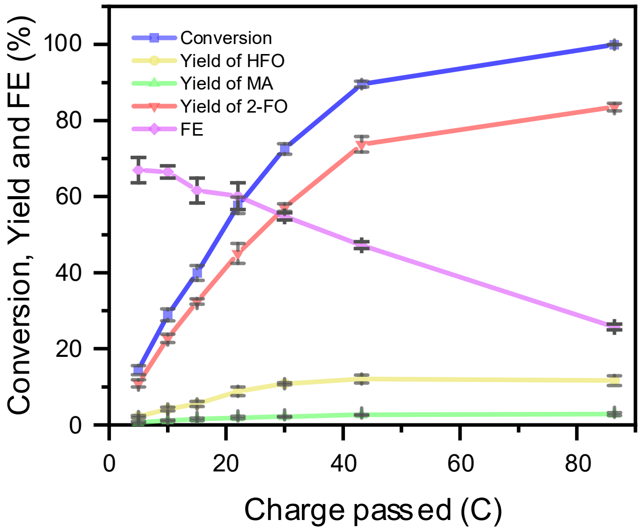

Fig. S2. The electrochemical oxidation of FA in pH 2. 10 mM FA at 1 mA constant current in pH 2 buffer at 80 °C with 0 – 86.4 C charge passed (24 h). WE: Pt, CE: Pt. Experiments were performed in triplicate and error bars correspond to the standard deviation of three independent measurements.

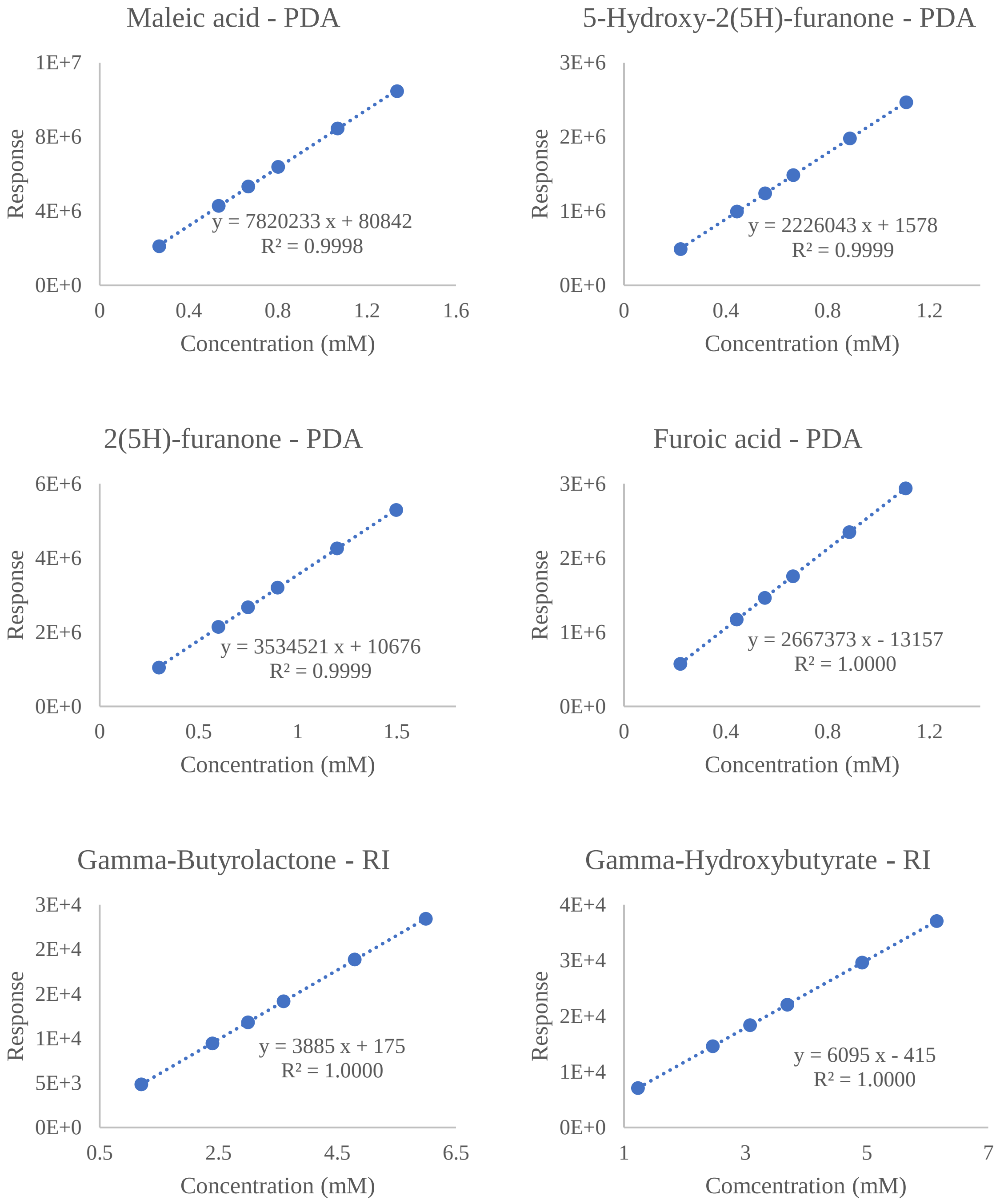

Fig. S3. HPLC calibration curves of different products. Obtained from the photodiode array (PDA) and refractive index (RI) detectors.

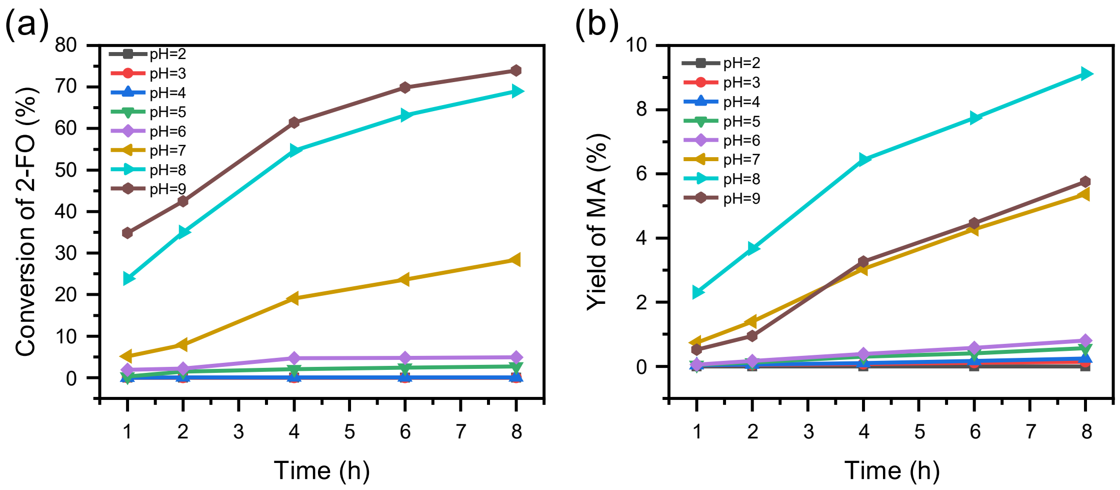

Fig. S4. pH stability examination of 2-FO in pH 2 to 9. **(a)** conversion of 2-FO and **(b)** yield of MA. Reaction conditions: 10 mM 2-FO in 10 mL different pH of 0.5 M potassium phosphate buffer at 80 °C.

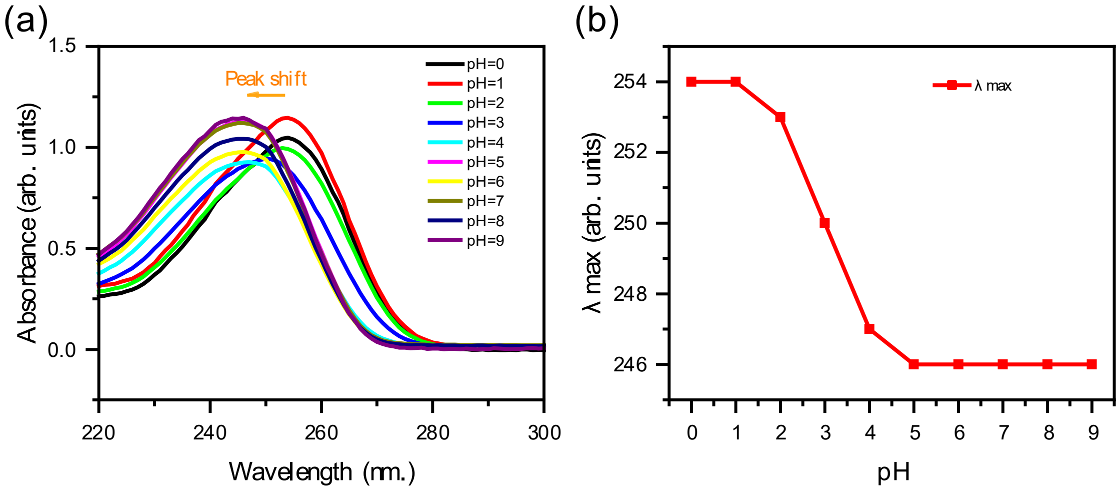

Fig. S5. (a) The UV-Vis absorbance of FA (0.1 mM) **(b)** The maximum UV-Vis absorbance (λ max) of FA from pH 2 to 9. The FA λ_{max} underwent a blue shift as the carboxyl group gradually deprotonated between pH 1 – 5, shifting the λ_{max} from 254 to 246 nm. A comparable λ_{max} shift could be seen with other aromatic acids.[11] The stable λ_{max} at pH before 1 and after pH 5 indicated that FA existed in FA molecular form and its anion form, respectively.

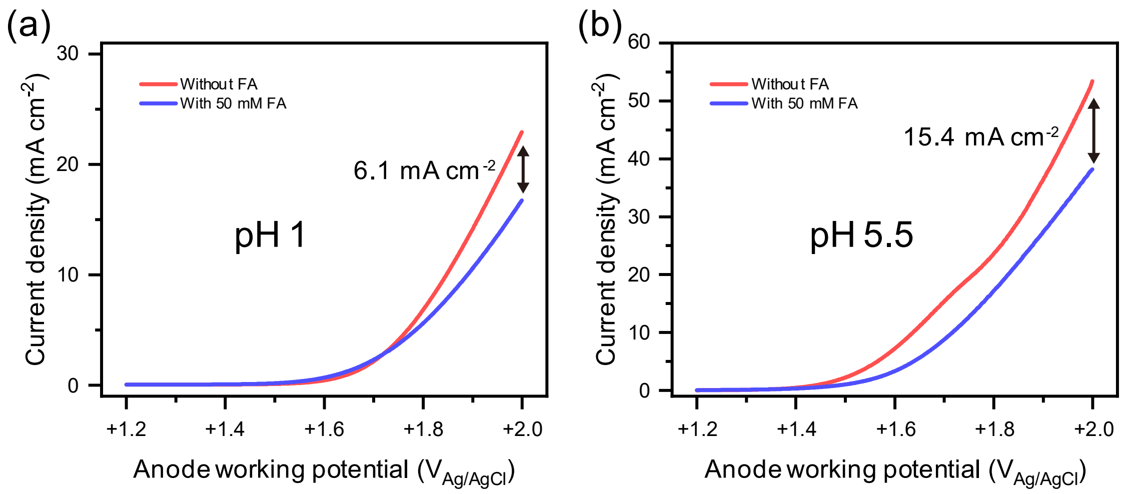

Fig. S6. LSV study at (a) pH 1 and (b) pH 5.5 with or without 50 mM FA. Reaction conditions: Scan rate 50 mV/s; 80 °C; WE: Pt; CE: Pt.

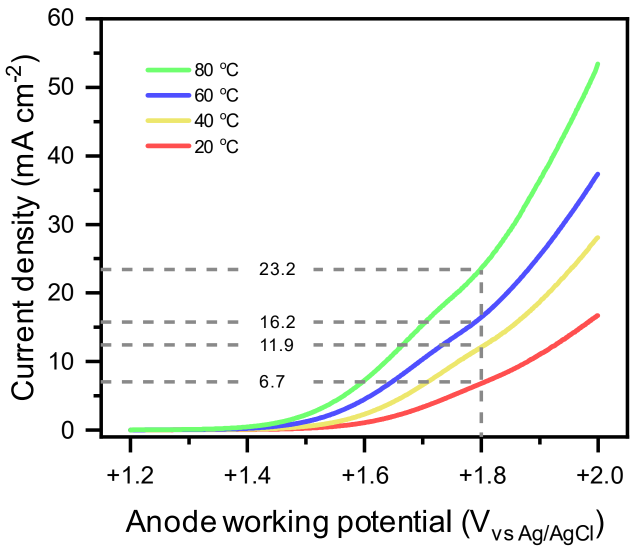

Fig. S7. Linear sweep voltammograms (LSVs) at 20 to 80 °C temperature range. Conditions: pH 5.5; without FA; WE: Pt; CE: Pt.

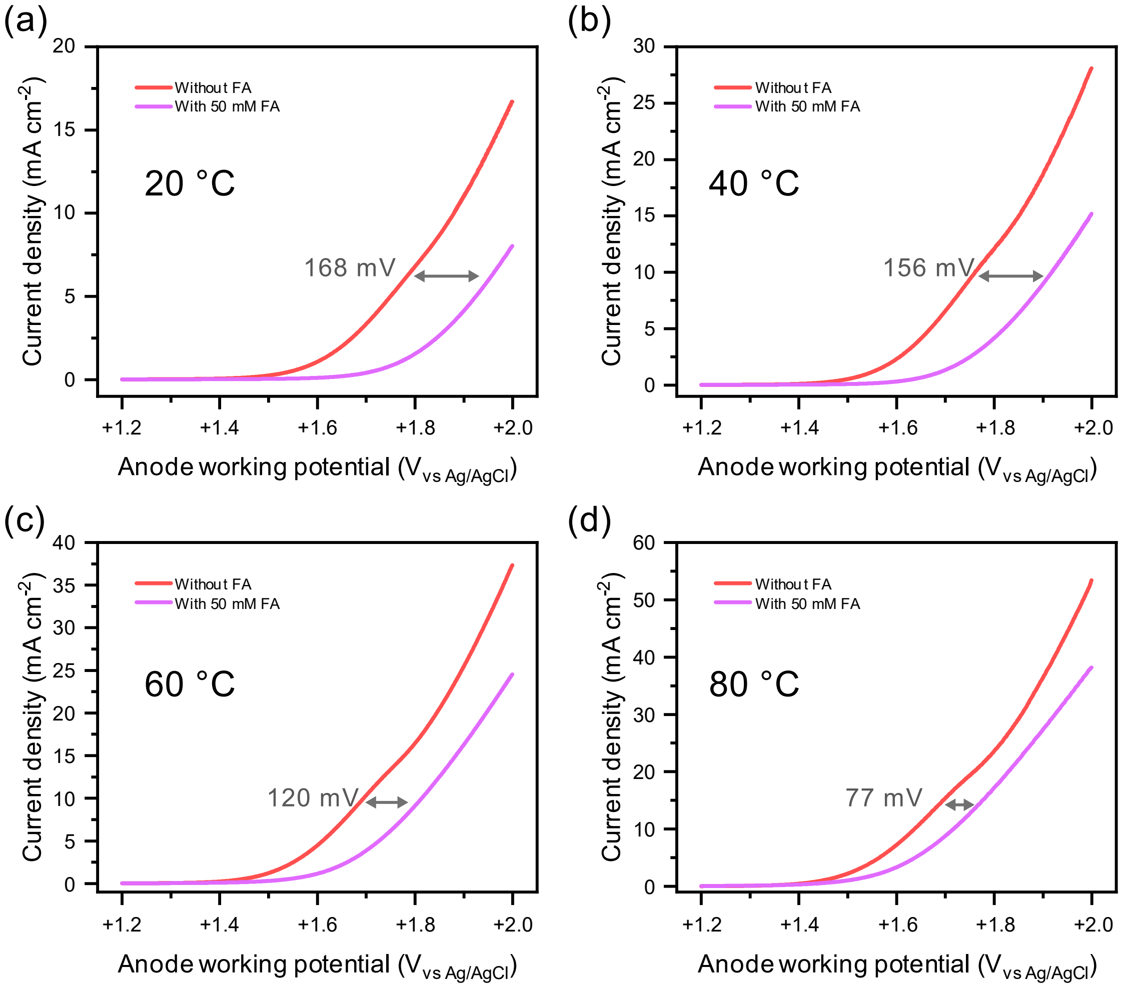

Fig. S8. Linear sweep voltammograms (LSVs) curve of the electrode in pH 5.5 buffer with and without 50 mM of FA at (a) 20 (b) 40 (c) 60 and (d) 80 °C, respectively. The potential differences were measured at j = 7.5 mA cm^{-2}. WE: Pt; CE: Pt.

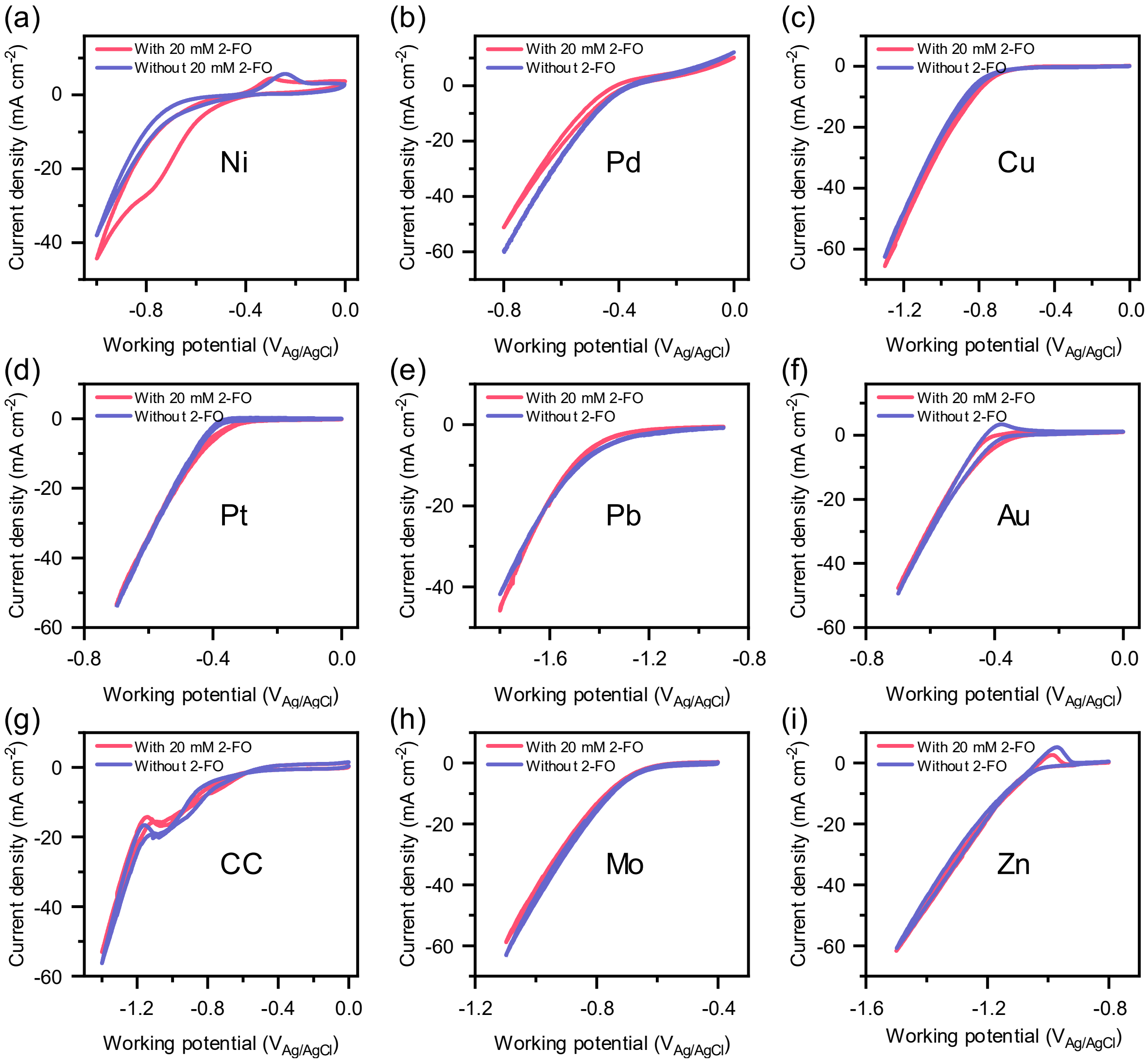

Fig. S9. Cyclic voltammograms (CVs) in the presence and absence of 20 mM of 2-FO in 20 mL pH 5.5 electrolyte at 80 °C using different working electrodes (a) Ni; (b) Pd; (c) Cu; (d) Pt; (e) Pb; (f) Au; (g) CC; (h) Mo; (i) Zn as WE, respectively.

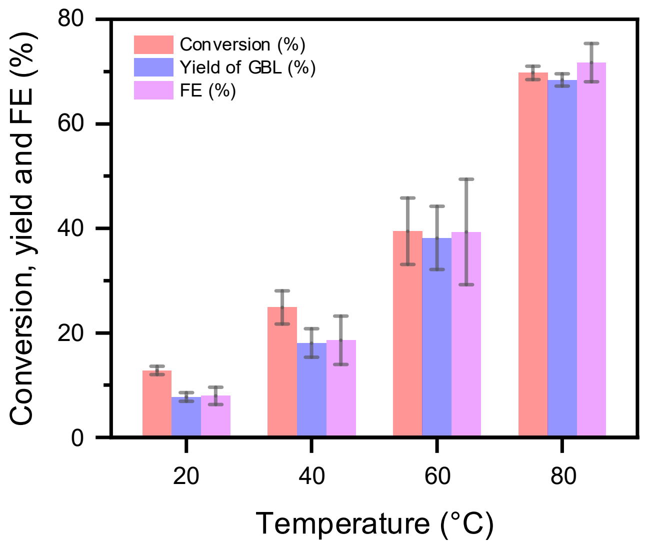

Fig. S10. The electrochemical reduction (ECH) of 2-FO at 20 – 80 °C. Experiments were performed in triplicate and error bars correspond to the standard deviation of three independent measurements. Reaction conditions: 20 mM 2-FO in 20 mL pH 5.5 buffer; 2.0 $V_{Ag/AgCl}$; WE: Pt, CE: Ni.

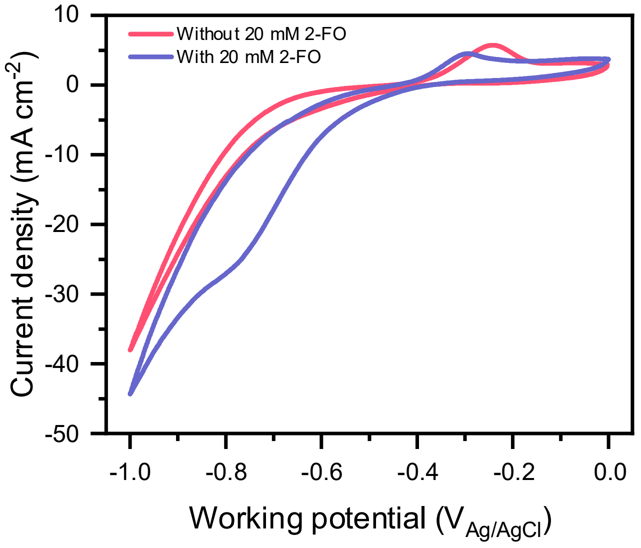

Fig. S11. Cyclic voltammograms (CVs): With and without 20 mM 2-FO in pH 5.5 electrolyte at 80 °C. WE: Ni; CE: Pt.

(a)

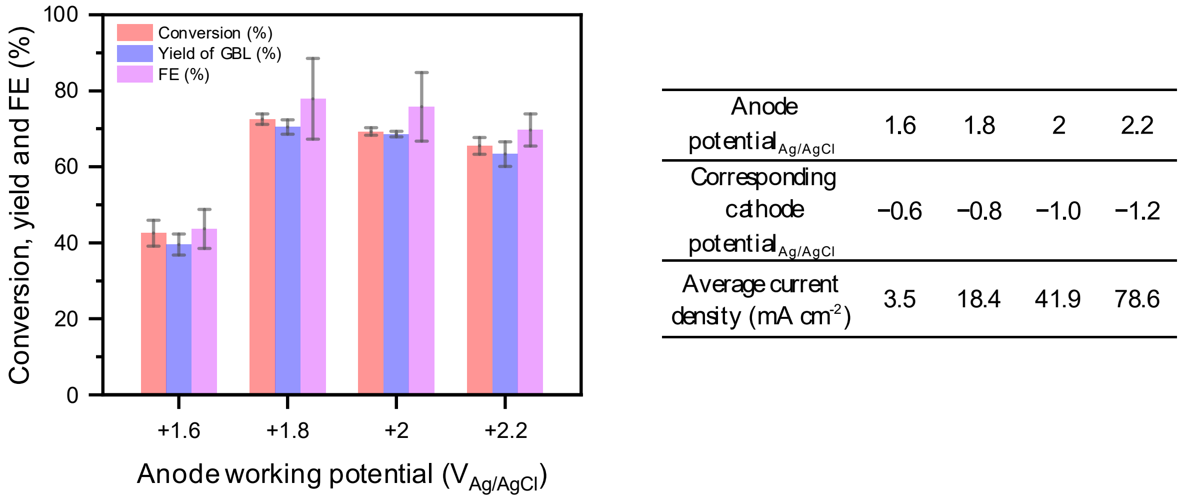

(b)

Anode potential$_{Ag/AgCl}$	1.6	1.8	2	2.2
Corresponding cathode potential$_{Ag/AgCl}$	−0.6	−0.8	−1.0	−1.2
Average current density (mA cm^{-2})	3.5	18.4	41.9	78.6

Fig. S12. (a) The electrochemical reduction (ECH) of 2-FO at an applied anodic potential of 1.6 to 2.2 $V_{Ag/AgCl}$ and (b) the corresponding cathode potential. Experiments were performed in triplicate and error bars correspond to the standard deviation of three independent measurements. Reaction conditions: 20 mM 2-FO in 20 mL pH 5.5 buffer at 80 °C; WE: Pt, CE: Ni.

Note: The objective was to examine whether the ECO and ECH reactions would operate in a comparable potential range suitable for their integration. We recorded the cathode potentials using a multimeter (measured between the cathode and reference electrode) while the electrochemical workstation monitored the working potentials for the anode (Fig. S12). The investigation began with the Pt operated at +1.6 $V_{Ag/AgCl}$, which was the lowest potential that generated a reasonable current flow at an average of ~3.5 mA cm^{-2}. The CV analysis revealed that +1.6 $V_{Ag/AgCl}$ was close to the onset potential of HER (Fig. S11). While the Pt operated at +1.6 $V_{Ag/AgCl}$, the Ni cathode working potential reached -0.6 $V_{Ag/AgCl}$ and yielded 38.3% GBL. As the Pt working potential increased to +1.8 and +2.0 $V_{Ag/AgCl}$, the Ni reduction potential raised to -0.8 and -1.0 $V_{Ag/AgCl}$, respectively, and the GBL respective yield were 70.5 ± 1.9 % and 68.6 ± 0.7 %. After that, at +2.2 V, the corresponding cathodic working potential became too high that favoured HER instead of ECH of 2-FO to GBL, and thus the GBL yield, and FE began to decline.

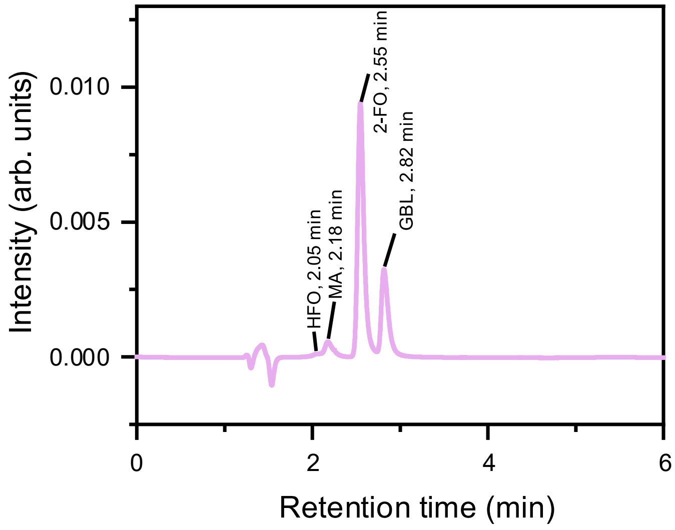

Fig. S13. HPLC chromatograms of PDA @ 210 nm sample analyses from the ECH of 20 mM 2-FO in 20 mL of 0.5 M pH 5.5 buffer at +2.0 $V_{Ag/AgCl}$, after 300-coulomb charge passed, referenced to the Pt anode paired with a Ni counter electrode at 80 °C. Note: MA: Maleic acid; HFO: 5-Hydroxy-2(5*H*)-furanone; 2-FO: 2(5*H*)-Furanone; GBL: Gamma-butyrolactone.

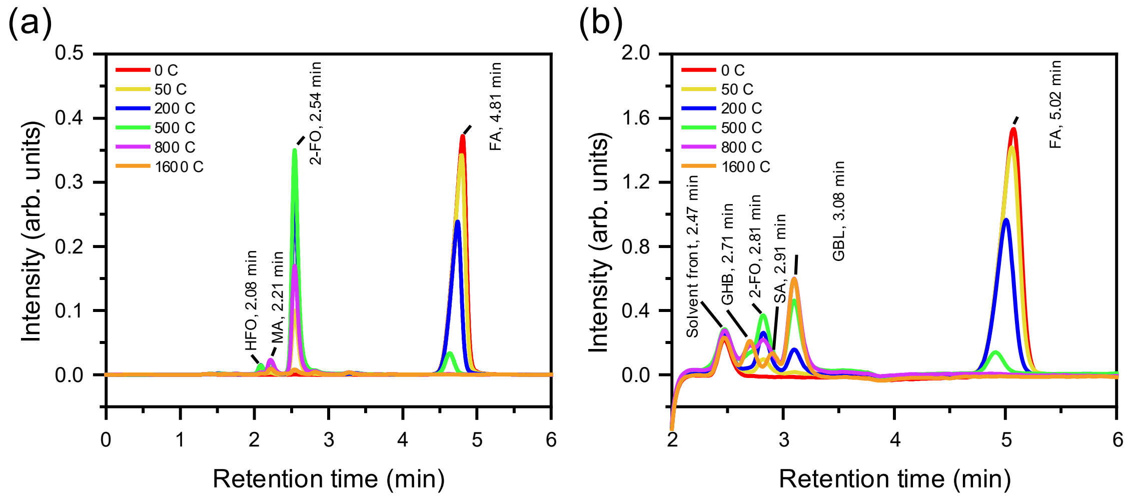

Fig. S14. HPLC chromatograms of (a) -PDA @ 210 nm and (b) -RI sample analyses collected periodically from a time-resolved electrolysis of 100 mM FA in 20 mL of 0.5 M pH 5.5 buffer at +2.0 $V_{Ag/AgCl}$ referenced to the Pt anode paired with a Ni counter electrode at 80 °C. Note: MA: Maleic acid; HFO: 5-Hydroxy-2(5H)-furanone; 2-FO: 2(5H)-Furanone; FA: Furoic acid; GBL: Gamma-Butyrolactone; GHB: Gamma-Hydroxybutyrate; SA: Succinic acid.

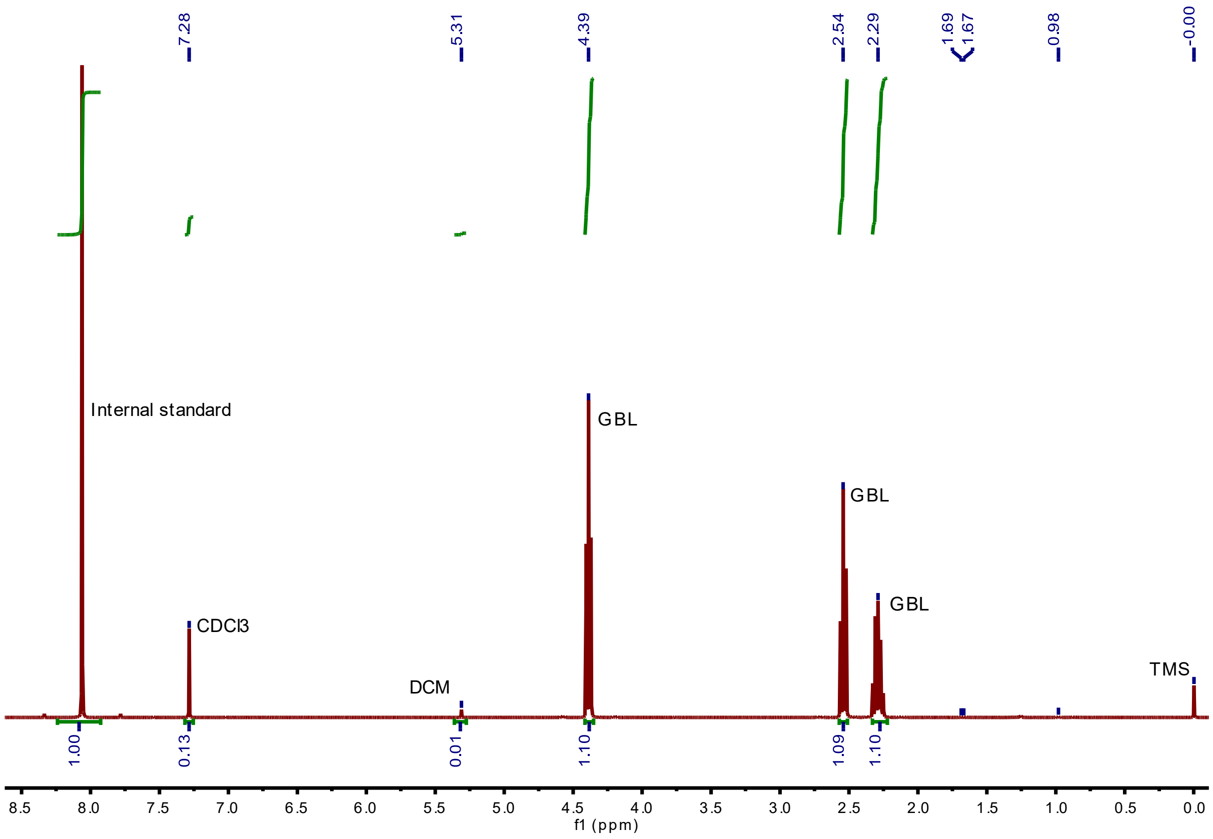

Fig. S15. The purity of GBL was examined by ^{1}H NMR. Reaction conditions: 50 mA cm^{-2}, 24 h, 5.6 g FA in 500 mL (100 mM) pH 5.5 buffer, 3*3 cm Pt as anode paired with 3*3 cm Ni as cathode, 80 °C. The solution was extracted by 500*2 mL of DCM. Then, the organic solution was rotary evaporated to obtain the high-purity GBL. Formic acid was used as an internal standard for quantitation.

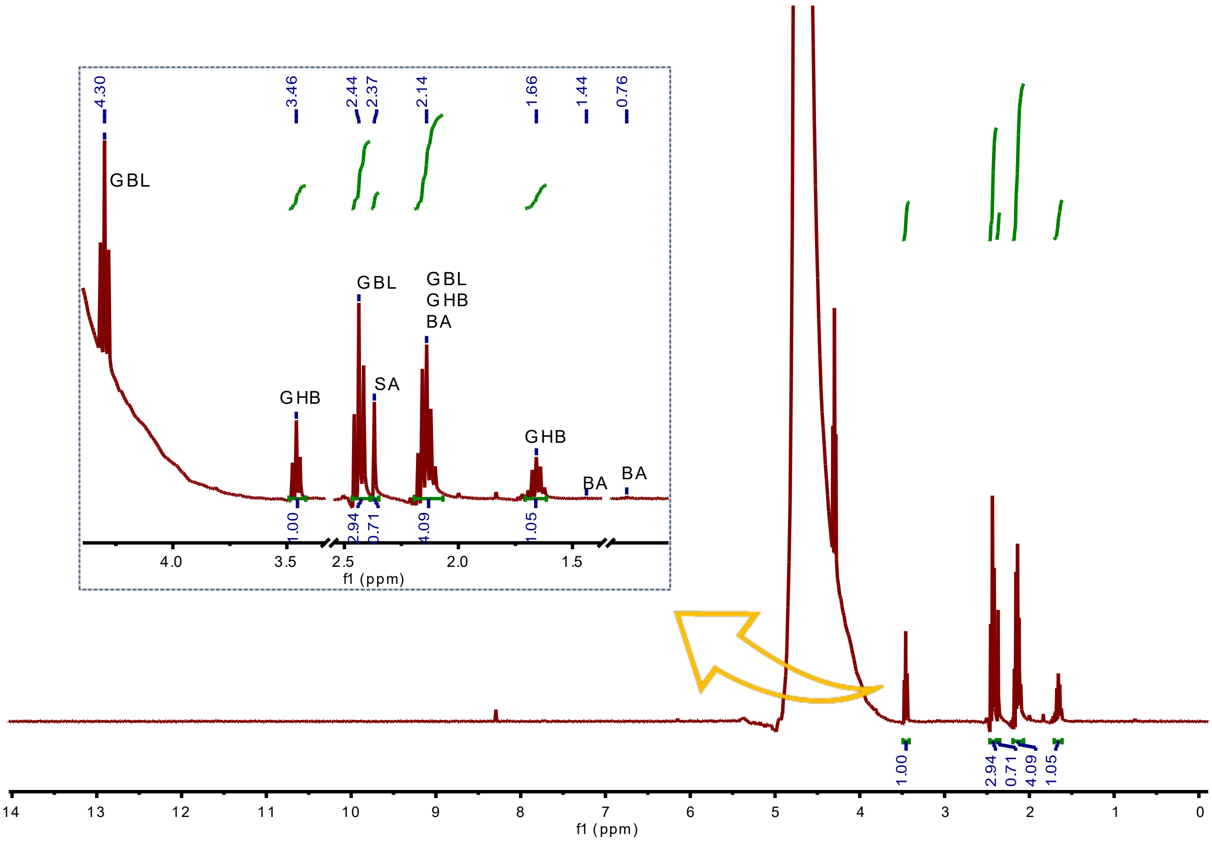

Fig. S16. ^1H NMR analysis of pre-extracted electrolyte in D_2O. Reaction conditions: 50 mA cm^{-2}, 24 h, 5.6 g FA in 500 mL (100 mM) pH 5.5 buffer, 3*3 cm Pt as anode paired with 3*3 cm Ni as cathode, 80 °C. Note: GBL: Gamma-Butyrolactone; GHB: Gamma-Hydroxybutyrate; SA: Succinic acid; BA: Butyric acid.

Supplementary References

1. Sodhi, R. K.; Paul, S.; Clark, J. H., A comparative study of different metal acetylacetonates covalently anchored onto amine functionalized silica: a study of the oxidation of aldehydes and alcohols to corresponding acids in water. *Green Chemistry* **2012,** *14* (6).
2. Gupta, N. K.; Fukuoka, A.; Nakajima, K., Metal-Free and Selective Oxidation of Furfural to Furoic Acid with an N-Heterocyclic Carbene Catalyst. *ACS Sustainable Chemistry & Engineering* **2018,** *6* (3), 3434-3442.
3. Nocito, F.; Ditaranto, N.; Linsalata, D.; Naschetti, M.; Comparelli, R.; Aresta, M.; Dibenedetto, A., Selective Aerobic Oxidation of Furfural into Furoic Acid over a Highly Recyclable MnO2@CeO2 Core–Shell Oxide: The Role of the Morphology of the Catalyst. *ACS Sustainable Chemistry & Engineering* **2022,** *10* (26), 8615-8623.
4. Yu, H.; Ru, S.; Dai, G.; Zhai, Y.; Lin, H.; Han, S.; Wei, Y., An Efficient Iron(III)-Catalyzed Aerobic Oxidation of Aldehydes in Water for the Green Preparation of Carboxylic Acids. *Angew Chem Int Ed Engl* **2016,** *56*, 3867-3871.
5. Wang, C.; Wu, Y.; Bodach, A.; Krebs, M. L.; Schuhmann, W.; Schüth*, F., A Novel Electrode for Value-Generating Anode Reactions in Water Electrolyzers at Industrial Current Densities. *Angew Chem Int Ed Engl* **2023,** *62*.
6. Zeng, Z.; Wu, S.; Huang, X.; Wei, Z., Electrochemical Oxidation of Furfural on NiMoP/NF: Boosting Current Density with Enhanced Adsorption of Oxygenates. *Small* **2023**, e2305462.
7. Zhong, Y.; Ren, R.-Q.; Wang, J.-B.; Peng, Y.-Y.; Li, Q.; Fan, Y.-M., Grass-like NixSey nanowire arrays shelled with NiFe LDH nanosheets as a 3D hierarchical core–shell electrocatalyst for efficient upgrading of biomass-derived 5-hydroxymethylfurfural and furfural. *Catalysis Science & Technology* **2022,** *12* (1), 201-211.
8. Ouyang, D.; Gao, D.; Hong, J.; Jiang, Z.; Zhao, X., Promoting and controlling electron transfer of furfural oxidation efficiently harvest electricity, furoic acid, hydrogen gas and hydrogen peroxide. *Journal of Energy Chemistry* **2023,** *79*, 135-147.
9. Shi, S.-S.; Zhang, X.-Y.; Zong, M.-H.; Wang, C.-F.; Li, N., Selective synthesis of 2-furoic acid and 5-hydroxymethyl-2-furancarboxylic acid from bio-based furans by recombinant Escherichia coli cells. *Molecular Catalysis* **2019,** *469*, 68-74.
10. Zheng, Z.; Xu, Q.; Tan, H.; Zhou, F.; Ouyang, J., Selective Biosynthesis of Furoic Acid From Furfural by Pseudomonas Putida and Identification of Molybdate Transporter Involvement in Furfural Oxidation. *Front Chem* **2020,** *8,* 587456.
11. Polewski, K.; Kniat, S.; Slawinska, D., Gallic acid, a natural antioxidant, in aqueous and micellar environment: spectroscopic studies. *Curr Top Biophys* **2002,** *26* (2), 217-227.
