# Supplementary material for: One-pot redox cascade paired electrosynthesis of gamma-butyrolactone from furoic acid
Source: Nat Commun. 2024 Feb 7;15:1141. doi: 10.1038/s41467-024-45278-z (PMC10850494; doi:10.1038/s41467-024-45278-z)
Supplement: Supplementary file 2 — Peer Review File [file 41467_2024_45278_MOESM2_ESM.pdf]

nature portfolio

Peer Review File

One-Pot Redox Cascade Paired Electrosynthesis of Gamma-Butyrolactone from Furoic Acid

Open Access This file is licensed under a Creative Commons Attribution 4.0 International License, which permits use, sharing, adaptation, distribution and reproduction in any medium or format, as long as you give appropriate credit to the original author(s) and the source, provide a link to the Creative Commons license, and indicate if changes were made. In the cases where the authors are anonymous, such as is the case for the reports of anonymous peer reviewers, author attribution should be to 'Anonymous Referee' followed by a clear attribution to the source work. The images or other third party material in this file are included in the article's Creative Commons license, unless indicated otherwise in a credit line to the material. If material is not included in the article's Creative Commons license and your intended use is not permitted by statutory regulation or exceeds the permitted use, you will need to obtain permission directly from the copyright holder. To view a copy of this license, visit http://creativecommons.org/licenses/by/4.0/.

REVIEWER COMMENTS

Reviewer #1 (Remarks to the Author):

In this study, a one-pot redox cascade electrosynthesis of gamma-butyrolactone (GBL) from furoic acid (FA) was developed by a Pt(+) | Ni(-) electrode pair in an undivided cell, which broadens the electrochemical paths to biomass-derived feedstock valorization. The authors have maken affords to demonstrate the feasibility of FA oxidation to 2(5H)-furanone (2-FO) by Pt electrode and 2-FO reduction to GBL by Ni electrode, respectively, under different pH environments and reaction temperatures. They combined the two redox reactions in one cell under the same condition. In the current version, however, the data is not sufficient, and the demonstration of the catalytic mechanism is not solid. In addition, the manuscript is not well prepared, many sentence errors are needed to be revised.

1. The authors need to provide detailed data for product quantification using HPLC, including the calibration curves of different products and the retention time curves of corresponding time-resolved electrolysis. The detection of trace products also requires convincing data.
2. It is highly suggested that the authors elaborate the reasons (mechanisms) for the high reaction selectivities of Pt and Ni electrode in corresponding electrode reactions, compared with other metal electrodes. It would be better to provide evidence, either theoretical or practical.
3. The following sentence errors and confusing expressions should be checked and revised.

(1) Line 50, "with formaldehyde 90-110 °C".
(2) Line 87, what's mean by "chemical significance"?
(3) Line 64, "but the required stoichiometric oxidants, such as H2O2 and peroxymonosulfate, in harsh reaction environments, which called for a greener catalytic approach". Where is the "predicate"?
(4) Line 104, "FA did not reduce electrochemically but only oxidized to 2-FO". It should be in the passive voice.
(5) Line 109, "transformation. (Fig. S1a)". The position of the parentheses should be checked throughout the manuscript.
(6) Line 137, "the electrode shifted from FA oxidation to oxygen evolution reaction (OER)".
(7) Line 147, "at 20, 40, 60, and 80 were".
(8) Line 154, "the OER onset potentials differences in the presence and absence of FA". How can the authors make sure the onset potentials are from OER?
(9) Line 270, "according to an earlier control experiment". Where is it?
(10) Line 292, "While the Pt operated at +1.6 VAg/AgCl, the Ni cathode working potential reached -0.6 VAg/AgCl". How can the authors determine the working potentials for anode and cathode simultaneously, with one reference electrode?
(11) Line 298, "between +1.8 – 2.0 VAg/AgCl".
(12) Line 304, "Based on our LC analysis". Where is it?
(13) Line 308, "all of which were detected". Evidence it in this work.
(14) Line 318, "+2.0 Pt-VAg/AgCl".

Reviewer #2 (Remarks to the Author):

Shengqin Liu et al describe paired electrosynthesis of gamma-butyrolactone (GBL) from furoic acid. GBL is very useful biobased platform chemical and as such the process has a high potential for manufacturing of this chemical on industrial scale. Regarding the scientific novelty - oxidation of furnoic acid to hydroxyfurane (a tautomer of the proposed anodic oxidation product (2(5H)-furanone) is known: see ref. 49 ACS Catal. 2019, 9, 10305. Consequently, the novelty of this article is pairing the oxidation with hydrogenation at nickel cathode which seems insufficient for publication in nature comm. and would be appropriate for more specialized journal.

Additional comments

1) Article contains unnecessary large amount of technical details which makes difficult to follow the essence.

2) For the optimization of both anodic process and cathodic process in unpaired mode the corresponding red or ox reactions should be provided. It can be assumed that for anodic oxidation a proton reduction to hydrogen is a cathodic process. It remains unclear what is anodic process for reduction of furanone (i.e. generation of hydrogen for this process)

3) The process has been performed on relatively small scale. 150 mL volume. The amount of product for this scale is not provided in the experimental description.

4) Minor comments:

a) The term “biochemical” typically refers to chemicals of living organisms, please replace

b) line 44. Critical solvent is not a correct term

c) line 112: “...the highest possible pH was still preferred to maximize FA deprotonation”I was not able to get sense of this sentence

d) line 188: what is meant by “b.p. of furan (radical)?

e) line 342: “It was also possible that part of the GBL yield was lost during the evaporative concentration” I guess it is easy to be determined with an internal standard

f) References need to include recent reviews of paired electrolysis

Reviewer #3 (Remarks to the Author):

This article illustrates an electrochemical path to make gamma butyrolactone (GBL) from furoic acid (FA) in a single electrochemical cell by performing oxidative and reductive steps on each electrode. FA is oxidized to 25H-furanone (2FO) at the anode, which is then reduced to GBL at the cathode. It is overall a clever scheme and the yields are notably high. I have a few significant concerns, but believe they might be addressable in a major revision.

1) Control experiments need to be explicitly shown. It needs to be shown that the elevated temperature with contact with the electrode metals does not cause any thermal catalytic reactions. CVs also need to be shown better illustrating the low activity of the opposite electrodes toward each reactant — i.e., please show 2FO being cycled into high anodic potential and FA being cycled to low reductive potential to clearly show their inertness toward side reactions at relevant conditions.

2) NMR data is only shown for the isolated product. Please provide raw electrolysis NMRs with identification of all peaks.

3) Is the 2,5H furanone actually pure or is there also 2,3H furanone?

4) The quoted selectivity should be based on conversion, not just detected products, as it makes it sound higher than it really is. All run data should also be compiled in an SI table (showing FE, carbon balance, Yield, reaction conditions etc). It is hard to synthesize all the information when relayed in paragraph form. Faradaic efficiency and carbon balance should also be commented on in addition to the yield/selectivity of the products in the abstract.

5) Some comparisons of OER onset are made with organic present and absent (Fig S5 and related comments). In the presence of organic, how is OER distinguished from the organic oxidation when only current is measured?

6) The formation of a furan "radical" is discussed, but this will not really be a radical if it is adsorbed to the surface. I would also change the phrasing to say this species is hypothesized — while the comments are plausible, there is no direct observation of this species.

7) Comments around page 18 were confusing with regard to the experiments to evaluate inertness of electrodes toward reacting with the wrong species — for example referring to positive voltages that cause HER (when it is meant that these push the cathode toward a more negative voltage to match the current, and that causes HER). Given my comment #1 about showing plain CV's in wider voltage ranges, that whole discussion may likely change anyway.

8) There are no error bars or reproduction of even the optimized conditions. How dependable are the yields, FE, CB etc?

9) It was stated that Pt was chosen because it is stable. It was also stated that polymers likely accumulate on the surface, meaning that the metal itself may not be very important. Do other stable electrodes like Au give different results?

10) Most biomass processing routes are going to lead to furfural (FF) as a starting point. It was found that the reactor concept in this paper doesn't work well with FF because it is too reactive with the cathode, and thus the authors start with FA instead. It is implied that pre-oxidation FF->FA will be very easy, but I think this needs more discussion and qualification about what realistic rates and selectivities would be achievable, and how the whole path would be integrated as a process.

Reviewer #1 (Remarks to the Author):

In this study, a one-pot redox cascade electrosynthesis of gamma-butyrolactone (GBL) from furoic acid (FA) was developed by a Pt(+) | Ni(-) electrode pair in an undivided cell, which broadens the electrochemical paths to biomass-derived feedstock valorisation. The authors have maken affords to demonstrate the feasibility of FA oxidation to 2(5H)-furanone (2-FO) by Pt electrode and 2-FO reduction to GBL by Ni electrode, respectively, under different pH environments and reaction temperatures. They combined the two redox reactions in one cell under the same condition. In the current version, however, the data is not sufficient, and the demonstration of the catalytic mechanism is not solid. In addition, the manuscript is not well prepared, many sentence errors are needed to be revised.

Response: Thank you for the positive and constructive feedback. We apologise that the submission draft did not contain sufficient data to clarify our scientific content. We have revised the manuscript substantially based on the reviewers' comments. We have also hired an external editing agency to check for any grammatical errors and typos.

1. The authors need to provide detailed data for product quantification using HPLC, including the calibration curves of different products and the retention time curves of corresponding time-resolved electrolysis. The detection of trace products also requires convincing data.

Response: Thank you for the suggestion. The detailed data on product quantification using HPLC, including the calibration curves of different products and the retention time curves of corresponding time-resolved electrolysis, is now included in the revised supporting information (SI). The evidence for the detection of trace products is also provided in the revised SI.

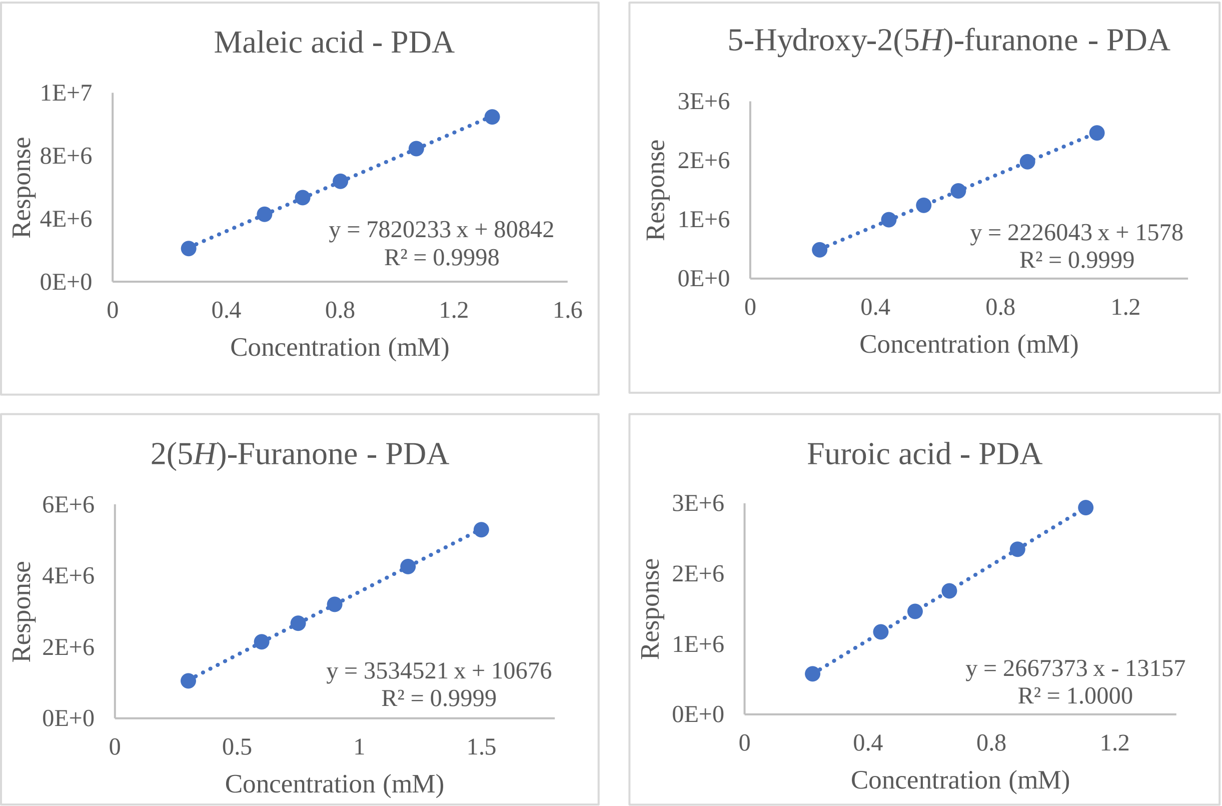

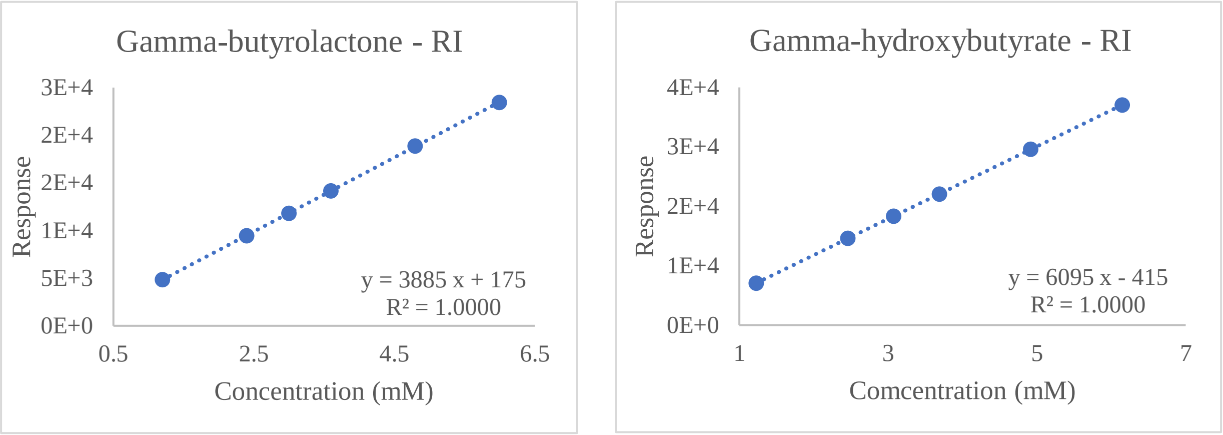

Fig. R1 (Fig. S3). HPLC calibration curves of different products obtained from the photodiode array (PDA) and refractive index (RI) detectors.

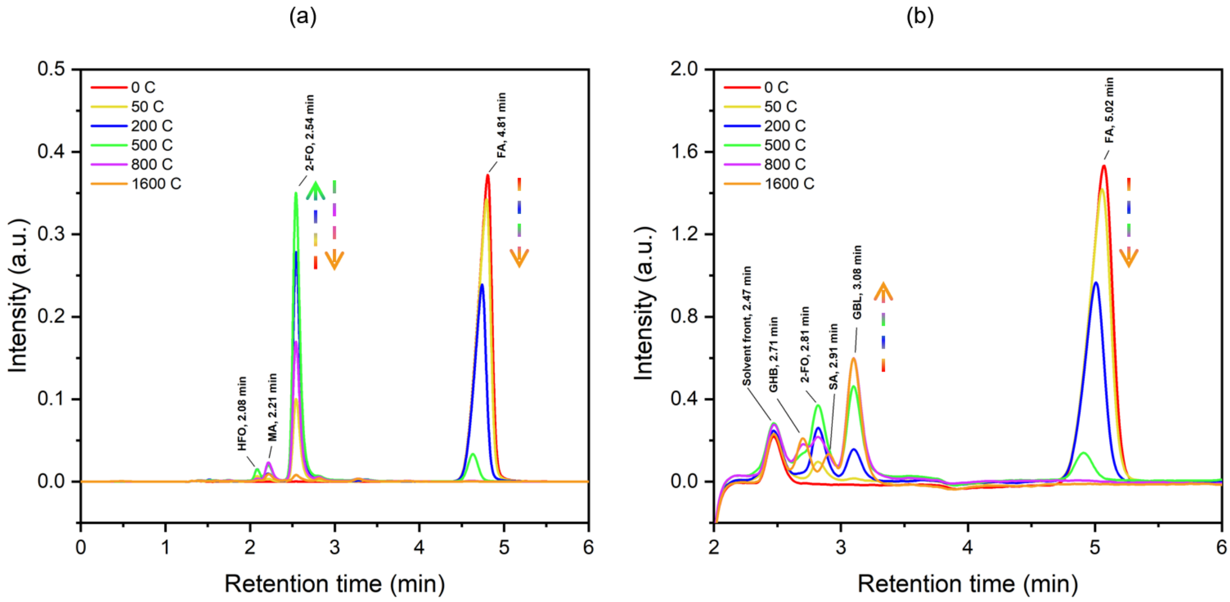

Fig. R2 (Fig. S14). HPLC chromatograms of (a) -PDA @ 210 nm and (b) -RI sample analyses collected periodically from a time-resolved electrolysis of 100 mM FA in 20 mL of 0.5 M pH 5.5 buffer at +2.0 $V_{Ag/AgCl}$ referenced to the Pt anode paired with a Ni counter electrode at 80 °C. Note: MA: Maleic acid; HFO: 5-Hydroxy-2(5*H*)-furanone; 2-FO: 2(5*H*)-Furanone; FA: Furoic acid; GBL: Gamma-butyrolactone; GHB: Gamma-hydroxybutyrate; SA: Succinic acid.

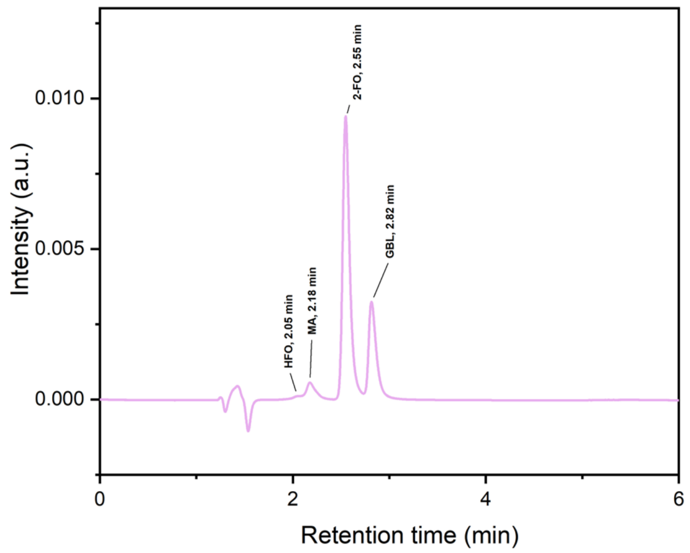

Fig. R3 (Fig. S13). HPLC chromatogram from PDA @ 210 nm showing trace production of MA and HFO, collected from the ECH of 20 mM 2-FO in 20 mL of 0.5 M pH 5.5 buffer at Pt +2.0 $V_{Ag/AgCl}$ paired with a Ni counter electrode after 300 coulomb charge passed at 80 °C. Note: MA: Maleic acid; HFO: 5-Hydroxy-2(5*H*)-furanone; 2-FO: 2(5*H*)-Furanone; GBL: Gamma-butyrolactone.

2. It is highly suggested that the authors elaborate the reasons (mechanisms) for the high reaction selectivities of Pt and Ni electrode in corresponding electrode reactions, compared with other metal electrodes. It would be better to provide evidence, either theoretical or practical.

Response: Thank you for raising this suggestion. We also agree with the reviewer that elaborating on the high reaction selectivity of Pt and Ni electrodes is necessary. We would also like to take the opportunity to clarify why Pt and Ni showed great electrocatalytic activities in their corresponding electrode reactions. Here, we define selectivity as the production of 2-FO instead of other over-oxidised or ring-opening products, such as maleic acid (MA), succinic acid (SA), or furanic polymer. Activity is defined as the catalytic performance of electrocatalytic oxidised FA.

a) Pt's high selectivity in producing 2-FO, contributed by temperature

The high selectivity observed from Pt (oxidising FA to 2-FO) is actually due to the reaction condition at 80 °C and mildly acidic condition. In our reaction, FA undergoes electrocatalytic oxidative decarboxylation to yield a furan radical intermediate. The 80 °C reaction temperature promotes its thermal desorption from the electrode surface, allowing it to react with the water to yield 2-FO (Fig. R4c). In our revised manuscript, we have added an EPR study (Fig. 1d) that captured the presence of a carbon-centred radical species, which we deduced to be the furan radical. At low temperatures, e.g., 20 °C, the furan radical retained well on the electrode and polymerised (Fig. R 4b), and thus produced more polymer on the Pt surface, which was observed in the ex-situ ATR-FTIR study (Fig. 1c).

b) Pt's high selectivity in producing 2-FO, contributed by pH

The mildly acidic environment at pH 5.5 was also important to enhance selectivity, as it promotes the deprotonation of FA, which can enhance anodic adsorption. The adsorption mode was supported by in-situ FTIR analysis and DFT calculations. [1,2] At pH 1, the conversion of FA reached 100%, but the 2-FO yield decreased to 28.3% ± 1.8% and the HFO yield increased to 14.7% ± 2.6% (from 11.6% ± 1.3% at pH 2) (Table R1). The decreased yield of 2-FO may be attributable to weak furan ring-directed adsorption of FA, which promoted its overoxidation and mineralisation (Fig. R4a). This would have increased the HFO yield and the oxidative mineralising activity, as indicated by the low carbon balance (CB). To solve this problem, we used a pH of 5.5, at which FA was nearly entirely deprotonated, as indicated by an ultraviolet-visible spectroscopy analysis (Fig. S5). At pH 5.5, the CB was 89.0% ± 1.6%, whereas at pH 1, it was only 46.1% ± 3.1% (Table R1). The carboxylate form of FA, i.e., furoate anion, tended to adsorb in a bidentate manner and upright orientation onto the Pt surface (Fig. R4c),[1,2] and promoted the ECO of the carboxyl group. The linear sweep voltammetry (LSV) analyses show a greater current density difference (with and without FA) at pH 5.5 than at pH 1, indicating a stronger furoate inhibition on Pt at pH 5.5, and thus confirming the enhancement of surface adsorption of the furoate anions at greater pH. (Fig. R5).

Table R1. (Table S1) Electrocatalytic oxidation of FA

Entry	WE	pH	Conversion (%)	Yield of products (%)			Sel of 2-FO (%)	CB (%)
				HFO	MA	2-FO		
1	Pt	1	100 ± 0.0	14.7 ± 2.6	3.2 ± 1.3	28.3 ± 1.8	28.3 ± 1.8	46.1 ± 3.1
2	Pt	5.5	88.9 ± 3.0	1.3 ± 0.5	3.0 ± 0.5	74.8 ± 4.4	84.2 ± 2.4	89.0 ± 1.6
3	Au	5.5	8.4 ± 3.6	0.8 ± 0.6	0.4 ± 0.2	6.5 ± 2.7	77.3 ± 4.0	92.2 ± 4.1

Reaction conditions: 10 mM FA in 10 mL of 0.5 M buffer at +1.8 $V_{Ag/AgCl}$ referenced to the WE (working anode) paired with a Pt counter electrode at 80 °C.

c) 2-FO production activity contributed by metal catalyst

In terms of activity, we compared the electrocatalytic oxidation performance of FA between Pt and Au. We found that using gold (Au) electrodes afforded a poor yield of 2-FO (6.5% ± 2.7%) (Table R1). Density functional theory (DFT) calculations suggested that the Au surface was more susceptible than the Pt surface to being 'poisoned' by upright furoate, thereby hindering furoate decarboxylation or oxidation steps.[3]

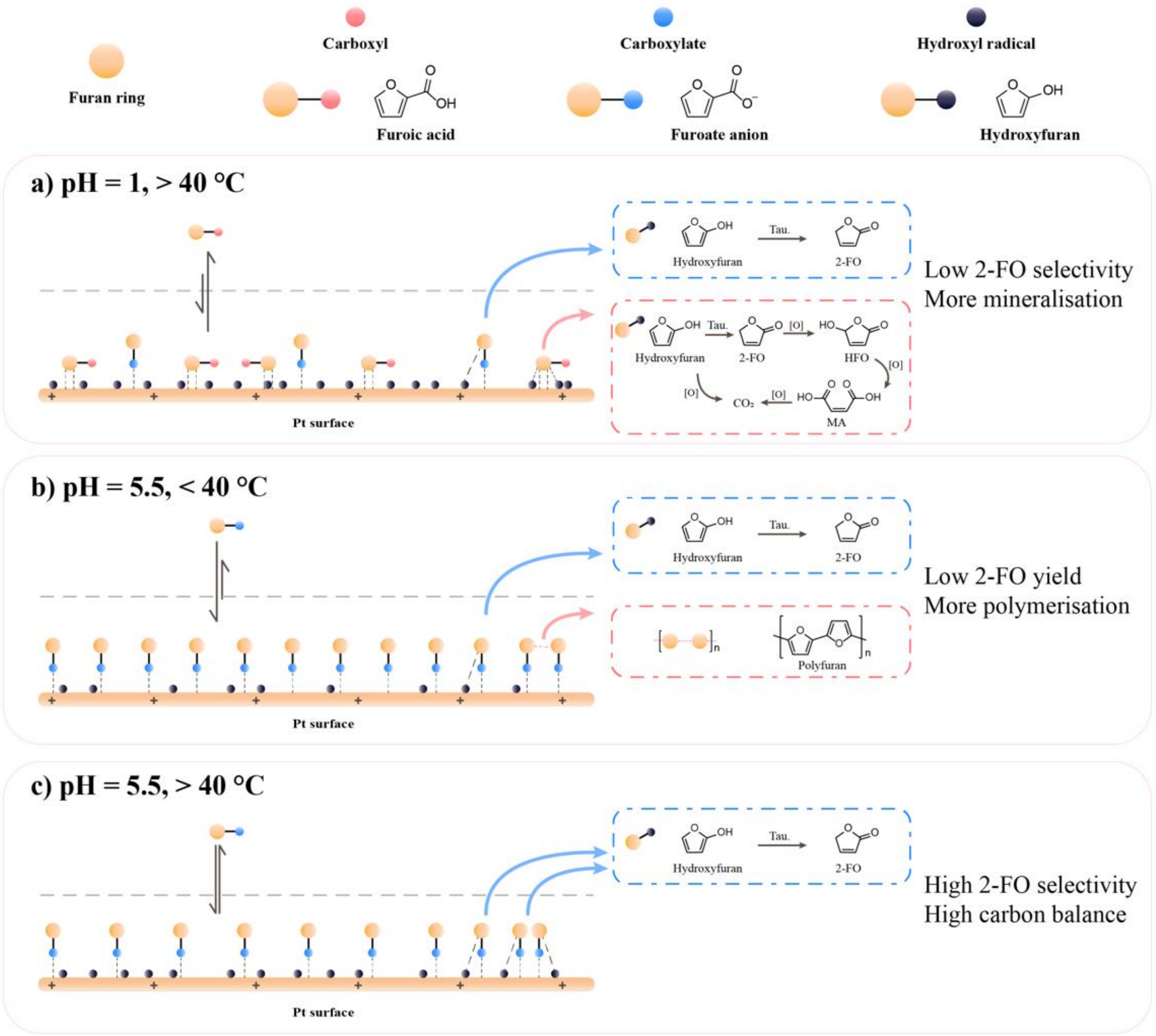

Fig. R4 (Fig. 2). Various anodic interface events at pHs 1 and 5.5 above and below 40 °C, respectively

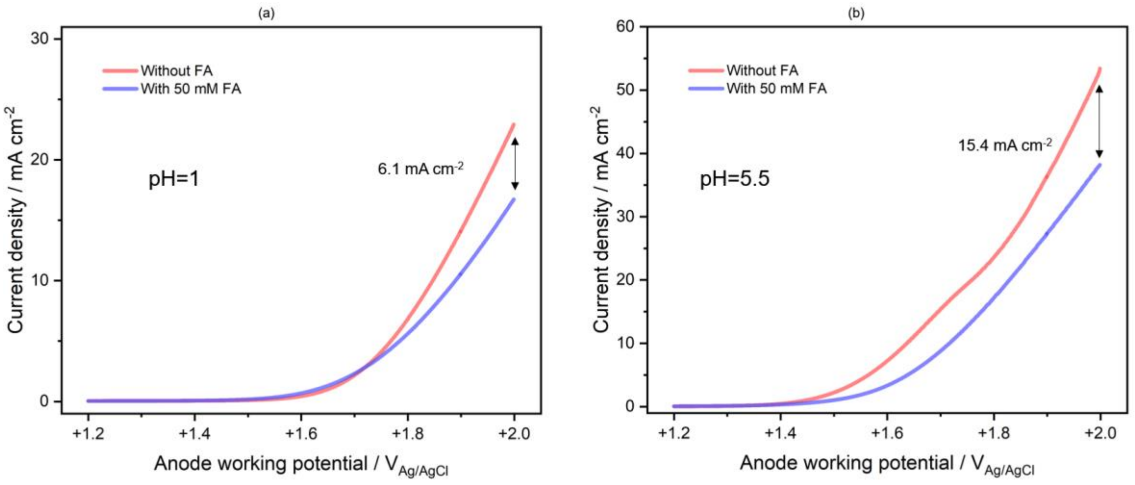

Fig. R5 (Fig. S6). LSV study at pH 1 and pH 5.5 with or without 50 mM FA. Reaction conditions: Scan rate 50 mV/s; 80 °C; WE: Pt; CE: Pt.

d) GBL production selectivity contributed by metal catalyst

The high C=C ECH efficiency can be explained by the d-band model devised by Nørskov et al.[4]. According to this model, a short distance between a d-band centre and a Fermi level enhances the binding energy between a metal surface and absorbents (Table R2). Among the top five most active metal catalysts, the binding energy of 2-FO followed the trend of Ni < Pd < Pt < Cu < Au, which closely aligned with the GBL yields observed in the experiment (Fig 3a). Delbecq et al.[5] reported that a decrease in the d-band width increased the interaction between the C=C bond and the metal surface, resulting in increased selectivity for C=C hydrogenation over C=O reduction. Ni possessed the narrowest d-band among the five metal catalysts, consistent with our experimental observation for C=C hydrogenation. To verify the electrocatalytic reduction activity for 2-FO hydrogenation, we performed CV analysis of all of the cathode materials (Fig. R6). Only the CVs of Ni displayed an observable difference in current with and without 2-FO, respectively, aligning with the aforementioned d-band width model of the C=C adsorption of 2-FO onto Ni. [Line 282 – 302]

Table R2. The *d*-band centre and width of different metal catalyst

	d-band centre (eV)	*d*-band width (eV)
Ni	**−1.29**	**3.0**
Pd	−1.83	4.1
Pt	−2.25	5.8
Cu	−2.67	3.0
Au	−3.56	5.7

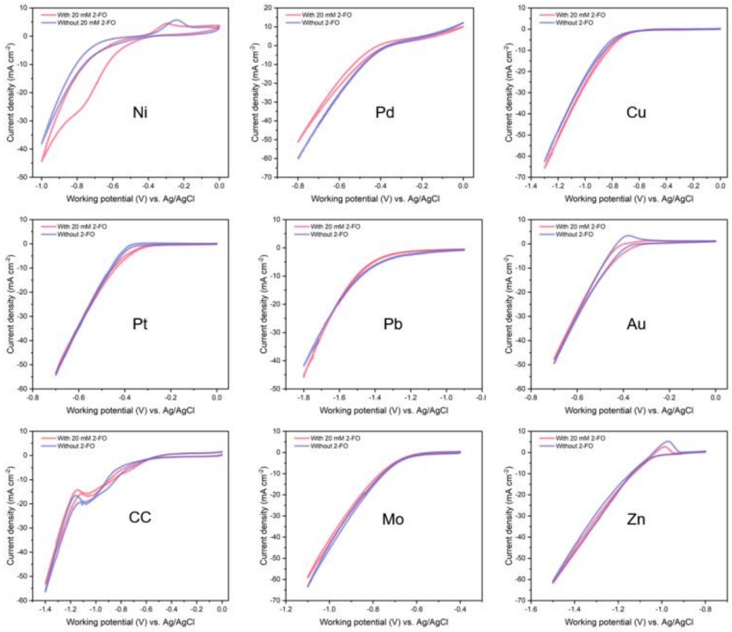

Fig. R6 (Fig. S9). Cyclic voltammograms (CVs) in the presence and absence of 20 mM of 2-FO in 20 mL pH 5.5 electrolyte at 80 °C using different working electrodes (a) Ni; (b) Pd; (c) Cu; (d) Pt; (e) Pb; (f) Au; (g) CC; (h) Mo; (i) Zn as WE, respectively.

3. The following sentence errors and confusing expressions should be checked and revised.
(1) Line 50, "with formaldehyde 90-110 °C".

Response: Thanks for your reminder. We have rewritten this sentence in [Line 52].

(2) Line 87, what's mean by "chemical significance"?

Response: Thank you for the question. Chemical significance meant "how did the chemical intermediate participate in the reaction?", as in what were their significant roles in this chemical reaction. We have changed this sentence to "We systematically investigated the influence of various reaction parameters on reactants and key intermediates to maximise the desired product selectivity from the respective electrodes and integrate redox reactions in an undivided cell." in the revised manuscript in [Line 107 – 110].

(3) Line 64, "but the required stoichiometric oxidants, such as H_2O_2 and peroxymonosulfate, in harsh reaction environments, which called for a greener catalytic approach". Where is the "predicate"?

Response: Thanks for your kind reminder. We have rewritten this sentence to "However, although these methodologies have provided valuable mechanistic insights into the conversion of FAL or FA to GBL, they require stoichiometric oxidants, such as H_2O_2 and peroxymonosulfate, and harsh reaction conditions." in the revised manuscript in [Line 68].

(4) Line 104, "FA did not reduce electrochemically but only oxidised to 2-FO". It should be in the passive voice.

Response: Thanks for the advice. We have rewritten this sentence to "Control experiments using cyclic voltammetry (CV) analysis and bulk electrolysis confirmed that FA could not be electrochemically reduced and could only be oxidised to 2-FO, which, in turn, could only be hydrogenated to GBL (Fig. S1)." in the revised manuscript in [Line 126 – 129].

(5) Line 109, "transformation. (Fig. S1a)". The position of the parentheses should be checked throughout the manuscript.

Response: Thanks for your suggestion. The whole manuscript has been revised with the suggested format.

(6) Line 137, "the electrode shifted from FA oxidation to oxygen evolution reaction (OER)".

Response: Thanks for the reminder. We rephrased this sentence to "because electrocatalytic oxidation shifted from FA oxidation to the oxygen evolution reaction (OER)" in the revised manuscript in [Line 172].

(7) Line 147, "at 20, 40, 60, and 80 were".

Response: Thank you for the reminder. We have revised the clause accordingly in the locations where it appears. [Line 177 and 190]

(8) Line 154, "the OER onset potentials differences in the presence and absence of FA". How can the authors make sure the onset potentials are from OER?

Response: Thank you for this question. We agree that in the presence of an organic substrate, part of the OER onset current is convoluted with organic oxidation. However, the Faradaic current resulted in organic oxidation is negligible compared to the OER because of the rapid scan rate of 50 mV s^{-1}, and the mass transport of fresh reductant, namely the FA, is relatively slow in the non-stirred environment of LSV experiments. Therefore, the observed sharp rise in current is OER. A comparable example measuring the OER onset potential change due to organic adsorptive inhibition (Fig. S8) is also commonly seen in the Kolbe electrolysis study, where volatile fatty acids adsorption shifted the OER onset potentials to more positive values.[6]

(9) Line 270, "according to an earlier control experiment". Where is it?

Response: Thank you for your reminder. It is in Fig. S4 and we have added "(Fig. S4)" at the end of this sentence in the revised manuscript in [Line 314].

(10) Line 292, "While the Pt operated at +1.6 $V_{Ag/AgCl}$, the Ni cathode working potential reached -0.6 VAg/AgCl". How can the authors determine the working potentials for anode and cathode simultaneously, with one reference electrode?

Response: Thank you for the question. We recorded the cathode potentials using a multimeter (measured between the cathode and reference electrode) while the electrochemical workstation monitored the working potentials for the anode. To make the manuscript concise, we moved the discussion of this part to the SI in Fig. S12.

(11) Line 298, "between +1.8 – 2.0 VAg/AgCl".

Response: Thank you for the kind reminder. We changed it to "between +1.8 – +2.0 $V_{Ag/AgCl}$" in the revised manuscript in [Line 331].

(12) Line 304, "Based on our LC analysis". Where is it?

Response: This was evidenced by HPLC and we quantified the concentration of GHB with 4.4 ± 0.7 % yield and added this data to Fig. 3e in the revised manuscript. GHB was produced through GBL ring-opening hydrolysis, a side reaction commonly at pH >2.[7]

(13) Line 308, "all of which were detected". Evidence it in this work.

Response: These overly oxidised products, such as HFO and MA were detected by HPLC (Fig. R7).

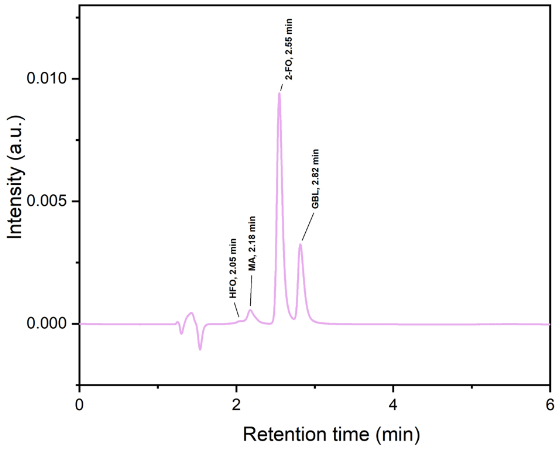

Fig. R7 (Fig. S13) HPLC chromatogram from PDA @ 210 nm showing trace production of MA and HFO, collected from the ECH of 20 mM 2-FO in 20 mL of 0.5 M pH 5.5 buffer at Pt +2.0 $V_{Ag/AgCl}$ paired with a Ni counter electrode after 300 coulomb charge passed at 80 °C. Note: MA: Maleic acid; HFO: 5-Hydroxy-2(5*H*)-furanone; 2-FO: 2(5*H*)-Furanone; GBL: Gamma-butyrolactone.

(14) Line 318, "+2.0 Pt-VAg/AgCl".

Response: Thanks for your reminder. In the revised manuscript, we changed this to "+2.0 $V_{Ag/AgCl}$ set to the Pt anode" in [Line 354].

Reviewer #2 (Remarks to the Author):

Shengqin Liu et al describe paired electrosynthesis of gamma-butyrolactone (GBL) from furoic acid. GBL is very useful biobased platform chemical and as such the process has a high potential for manufacturing of this chemical on industrial scale. Regarding the scientific novelty - oxidation of furnoic acid to hydroxyfurane (a tautomer of the proposed anodic oxidation product (2(5H)-furanone) is known: see ref. 49 ACS Catal. 2019, 9, 10305. Consequently, the novelty of this article is pairing the oxidation with hydrogenation at nickel cathode which seems insufficient for publication in nature comm. and would be appropriate for more specialised journal.

Response: Thank you for the constructive comments. We are excited about contributing a green and convenient electrocatalytic procedure to transform FA directly to GBL, and we hope this method can strengthen the role of biorefinery. In addition to enhancing the mechanism discussion, we increased the electrolysis scale to 5.6 g of FA and produced over 2 grams of 98+% pure GBL. We agree with the reviewer that there are sophisticated mechanism works published on the oxidation of furfural or furoic acid, and some of these works were helpful to our manuscript. However, the novelty of this work lies beyond just pairing the FA oxidation with a Ni-catalysed ECH of 2-FO. Please allow us to summarise the novelties here:

First, the electrocatalytic oxidation of FA to 2-FO and reduction of 2-FO to GBL occurs in a single undivided cell. And our reaction does not require a mediator, such as TEMPO, organic co-solvent, or a separator. To the best of the authors' knowledge, undivided paired electrolysis is rare, as most paired electrolysis requires an ion-exchange membrane separator to prevent redox crossover. Moreover, the undivided cell setting prevents pH fluctuations commonly observed in divided cells. In our case, if we employ a divided cell and the anodic pH gets too low, the FA oxidation product selectivity will shift from 2-FO to HFO, which prevents the formation of GBL.

Second, the high selectivity of FA to 2-FO was possible because of the discovery of thermal-dependent surface desorption of the furan radical from the electrode's surface and the pH-dependent adsorption state of the furoate anion. Such a discovery was not reported in any of the furfural or FA-focused electrochemical studies. To the best of the authors' knowledge, such a simple yet effective strategy to generate 2-FO and GBL is unprecedented in the electrosynthesis community.

Third, the highly efficient reduction of 2-FO to GBL was also achieved with 98.5% ± 0.5% olefin hydrogenation selectivity in an ambient condition, which mitigates the use of a high-pressure reaction apparatus. Furthermore, the one-pot reaction was scaled up to 5.6 g of FA and produced 2.1 ± 0.2 g of 98.1% ± 0.4% pure GBL, which was obtained through simple solvent extraction. There was no unintended redox reaction for the 2-FO and GBL. The appropriate inertness of 2-FO and FA is one of the key criteria that enables direct production of GBL from FA.

Fourth, earlier reported works mainly focused on oxidizing furfural or furoic acid to ring-opening products and their mechanisms. We focused on designing the reaction to halt at 2-FO instead of going further to maleic or succinic acids. We did so by designing the reaction conditions, such as pH and temperature, that favored the formation and retention of 2-FO. We also identified a suitable cathodic material that can rapidly hydrogenate the 2-FO into GBL. Thanks to the reviewers' comments, we have revised the manuscript substantially and hope this matches the standard of the reviewer and that of Nature Communications.

Additional comments

1) Article contains unnecessary large amount of technical details which makes difficult to follow the essence.

Response: Thank you for your suggestions. To improve the clarity of the manuscript, we have revised the manuscript content substantially and moved some discussions e.g., the discussion on 2-FO reduction at different applied voltages and some of the temperature-relevant work to the SI.

2) For the optimisation of both anodic process and cathodic process in unpaired mode the corresponding red or ox reactions should be provided. It can be assumed that for anodic oxidation a proton reduction to hydrogen is a cathodic process. It remains unclear what is anodic process for reduction of furanone (i.e. generation of hydrogen for this process)

Response: Thank you for asking this question. The revised SI now provides a discussion on the corresponding redox reactions.

During the reduction of 2-FO (Eq. 1), the hydrogen evolution reaction (Eq. 2) occurred in parallel on the cathode as well. As for the anodic processes, water splitting (Eq. 3) was the sole reaction since 2-FO could not be oxidised as control experiments verified. The electrochemical equations are as follows:

Cathode

2-FO + $2H^+ + 2e^-$ --> GBL Eq. 1

$2H^+ + 2e^-$ --> H_2 Eq. 2

Anode

H_2O --> $O_2 + 4H^+ + 4e^-$ Eq. 3

During the FA oxidation study in unpaired mode, furan-2-carboxylate anion (FA anion) underwent a single-electron oxidation to induce decarboxylation (Eq. 4) to become a furan radical, which reacts with water to yield 2-FO. (Eq. 5) The cathodic reaction would be a hydrogen evolution reaction. (Eq. 2) The electrochemical equations are as follows:

Anode

Furan-2-carboxylate ---> Furan radical + $CO_2 + e^-$ Eq. 4

Furan radical + H_2O --> 2-FO + $H^+ + e^-$ Eq. 5

Cathode

$2H^+ + 2e^-$ --> H_2 Eq. 2

3) The process has been performed on relatively small scale. 150 mL volume. The amount of product for this scale is not provided in the experimental description.

Response: Thanks for your advice. We have provided a more detailed description in the "*Scaled-up Electrolysis and Product Isolation*" section. We expanded the reaction scale to 5.6 grams of FA in 500 mL (100 mM) and obtained a 47.8 ± 3.5 % isolated yield of GBL with 98.1% ± 0.4% purity (Fig. R8).

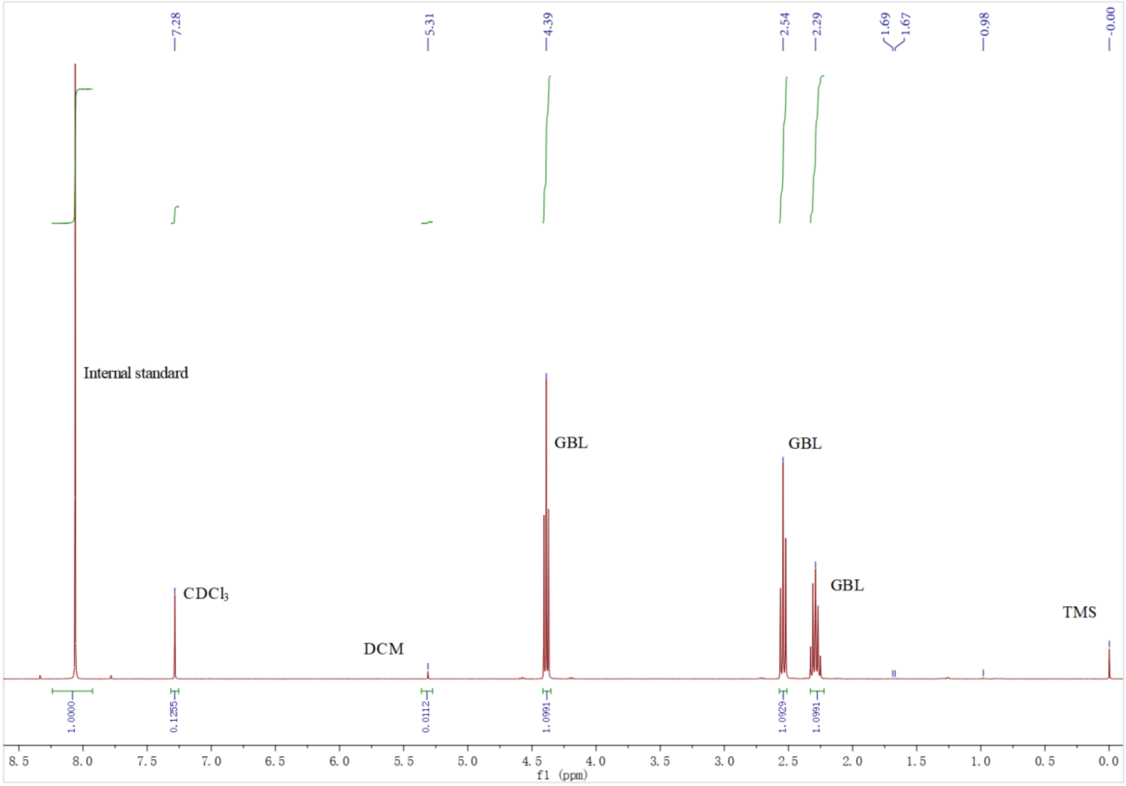

Fig. R8 (Fig. S15). The purity of GBL was examined by ^{1}H NMR. Reaction conditions: 50 mA cm^{-2}, 24 h, 3 × 3-cm Pt anode paired with 3 × 3-cm Ni cathode, pH 5.5, 80 °C. The solution was extracted by 500*2 mL of DCM. Then the organic solution was rotary evaporated to obtain the high-purity GBL. Formic acid was used as an internal standard for quantitation.

4) Minor comments:

a) The term "biochemical" typically refers to chemicals of living organisms, please replace

Response: Thanks for your suggestion. We changed it to "bio-derived chemicals" in [Line 43] in the revised manuscript.

b) line 44. Critical solvent is not a correct term

Response: Thanks for your reminder. We changed it to "non-toxic solvent" in [Line 45].

c) line 112: "...the highest possible pH was still preferred to maximise FA deprotonation" I was not able to get sense of this sentence

Response: Thanks for your question. We meant that the reaction should be performed at the highest possible pH, where FA is fully deprotonated. This is because 1) although the FA oxidation could work in an acidic environment between 2 – 5.5, pH 5.5 is preferred because that's when all the FA is deprotonated. The deprotonated FA is beneficial to promoting their surface adsorption. 2) a higher pH also benefits the subsequent ECH of 2-FO. We have updated the writing as follows:

"To solve this problem, we used a pH of 5.5, at which FA was nearly fully deprotonated, as indicated by an ultraviolet-visible spectroscopy analysis (Fig. S5)." [Line 144–146]

d) line 188: what is meant by "b.p. of furan (radical)?

Response: Thanks for your question. The "b.p. of furan (radical)" means the boiling point of furan radical. We have revised it with its full name in the revised manuscript in [Line 239].

e) line 342: "It was also possible that part of the GBL yield was lost during the evaporative concentration" I guess it is easy to be determined with an internal standard

Response: Thank you for your advice. GBL yield loss could happen in every step of the separation process, e.g., extraction, drying of the organic extract and evaporation. Due to the difference in boiling point, solubility, stability and the special peak shift for NMR spectra, it is hard to find an appropriate internal standard. After we quantified the GHB yield, we found the main loss of GBL was due to the hydrolysis of GBL to GHB during the long-time heating. Thus, to get a higher yield of GBL, we could potentially use a larger electrode operating at a higher current to shorten reaction time to reduce loss due to GBL hydrolysis to GHB. The development of a large-scale electrolysis system is currently underway.

f) References need to include recent reviews of paired electrolysis

Response: Thank you for your suggestion. It is meaningful to cite some recent reviews of paired electrolysis. And we added the following sentences to the *introduction* section from [line 82 to 92] in the revised manuscript.

In recent years, paired electrolysis has attracted increasing interest due to its potential application in advanced electro-synthetic routes, and enhanced energy efficiency and economic value. Typically, most paired electrolysis setups involve separating redox reactions using an ion-exchange membrane or a porous frit to minimise yield loss from reverse reactions. The product streams are then collected separately or combined, depending on specific reaction requirements.[8,9] Separator-free one-pot paired electrolysis setups are relatively rare because they require careful substrate selection and precise condition tuning to suppress reverse reactions. Thus, only a few such setups have been reported and have used elaborate electrochemical mediators and had limited current flow, hindering the achievement of high yields and efficiency.[10]

Reviewer #3 (Remarks to the Author):

This article illustrates an electrochemical path to make gamma butyrolactone (GBL) from furoic acid (FA) in a single electrochemical cell by performing oxidative and reductive steps on each electrode. FA is oxidised to 25H-furanone (2FO) at the anode, which is then reduced to GBL at the cathode. It is overall a clever scheme and the yields are notably high. I have a few significant concerns, but believe they might be addressable in a major revision.

Response: Thank you for providing such positive and constructive comments. We have revised the manuscript substantially and hope our revision can address the reviewer's concerns.

1) Control experiments need to be explicitly shown. It needs to be shown that the elevated temperature with contact with the electrode metals does not cause any thermal catalytic reactions. CVs also need to be shown better illustrating the low activity of the opposite electrodes toward each reactant — i.e., please show 2FO being cycled into high anodic potential and FA being cycled to low reductive potential to clearly show their inertness toward side reactions at relevant conditions.

Response: Thank you for this suggestion. We added a new series of LSV and bulk electrolysis control experiments, and they showed that no conversion occurred when we added the airflow in an open-circuit to different anode materials (Pt, Au) for the FA oxidation, and separately, adding H_2 to all kinds of cathode materials for the 2-FO reduction at 80 °C. CVs for opposite electrodes towards "the wrong" reactant were shown in Fig. R9. Both CVs show almost no difference in the absence and presence of the "wrong reactant", e.g. Pt for the oxidation of 2-FO and Ni for the reduction of FA, verifying the low activity of the opposite electrodes towards each other reactant.

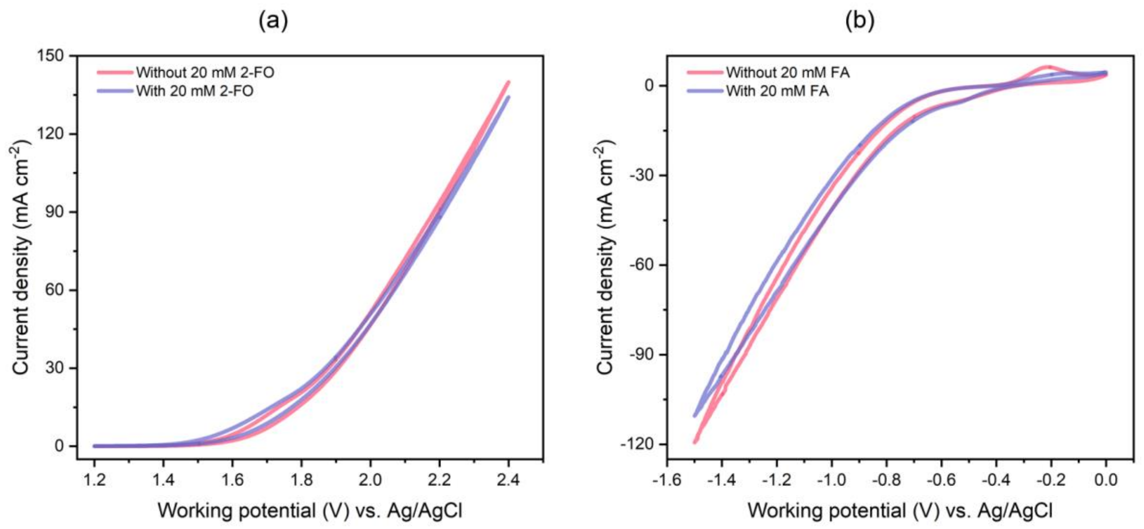

Fig. R9 (Fig. S1). Cyclic voltammograms (CVs): (a) Positive scan using Pt as working electrode with and without 20 mM 2-FO and (b) Negative scan using Ni as working electrode with and without 20 mM FA in 20 mL pH 5.5 electrolyte at 80 °C, respectively.

2) NMR data is only shown for the isolated product. Please provide raw electrolysis NMRs with identification of all peaks.

Response: Thanks for your suggestion. We added a pre-extracted ^{1}H NMR prepared from diluting our aqueous reaction sample with D_2O. GHB and succinic acid (SA) were also detected in small quantities. Based on ^{1}H-NMR integration, the GHB and SA yields were 12 % and 5% yield, respectively (Fig. R10). SA might be generated from the ring-opening of 2(3*H*)-furanone, which yields 4-oxobutanoic acid, followed by the ECO of its aldehyde group.[2] It may also be formed from the ECH of the C=C bond of MA, which came from HFO described in Scheme 2. A trace of butyric acid (BA) was also observed, which might be generated from the reduction of GHB.

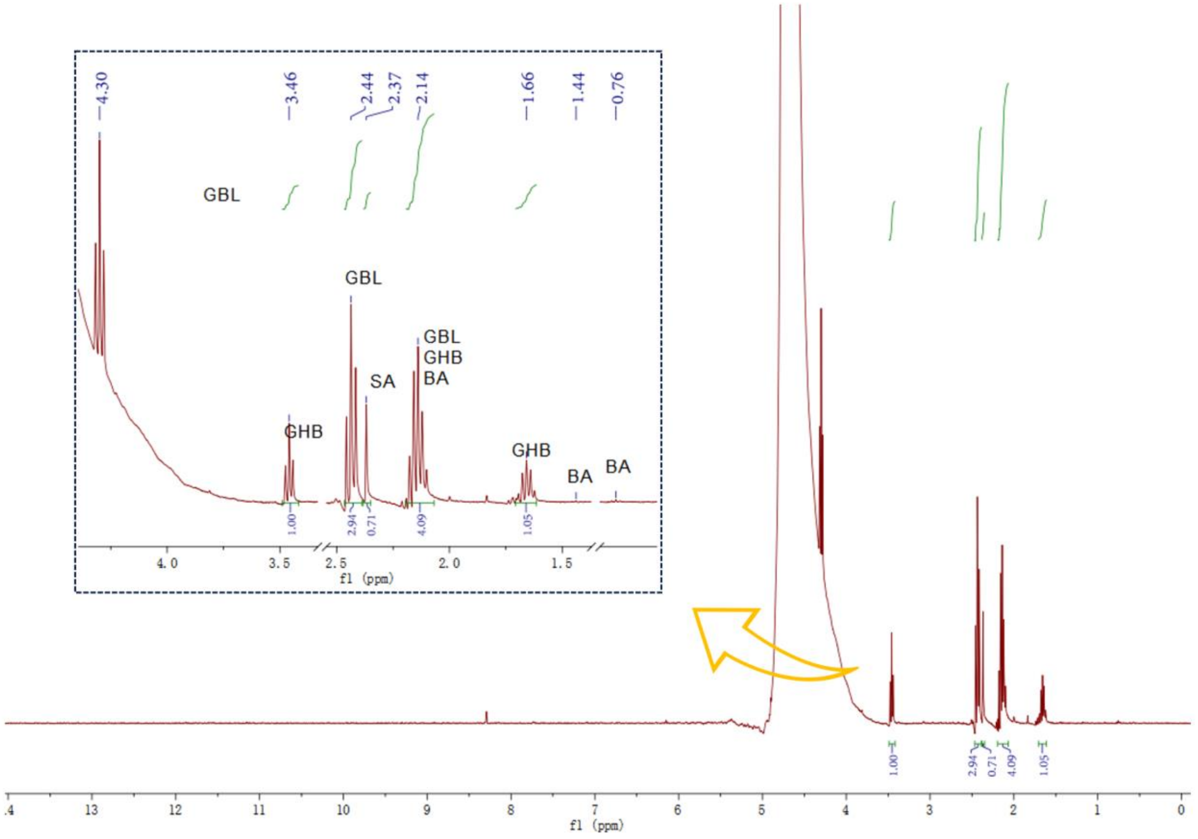

Fig. R10 (Fig. S16). ^{1}H NMR analysis of pre-extracted electrolyte in D_2O. Reaction conditions: 50 mA cm^{-2}, 24 h, 3 × 3-cm Pt anode paired with 3 × 3-cm Ni cathode, pH 5.5, 80 °C. Note: GBL: Gamma-Butyrolactone; GHB: Gamma-Hydroxybutyrate; SA: Succinic acid; BA: Butyric acid.

3) Is the 2,5H furanone actually pure or is there also 2,3H furanone?

Response: According to the raw reaction solution ^{1}H NMR result below (Fig. R11), there is no apparent 2(3*H*)-furanone (3-FO) peaks at 3.10, 5.52 and 6.74[11], indicating that the 2(5*H*)-furanone is pure after the experiment. However, we still believe 3-FO was formed during the electrolysis because succinic acid, which is the ring-opening product from 3-FO as Q2 discussed, was detected.

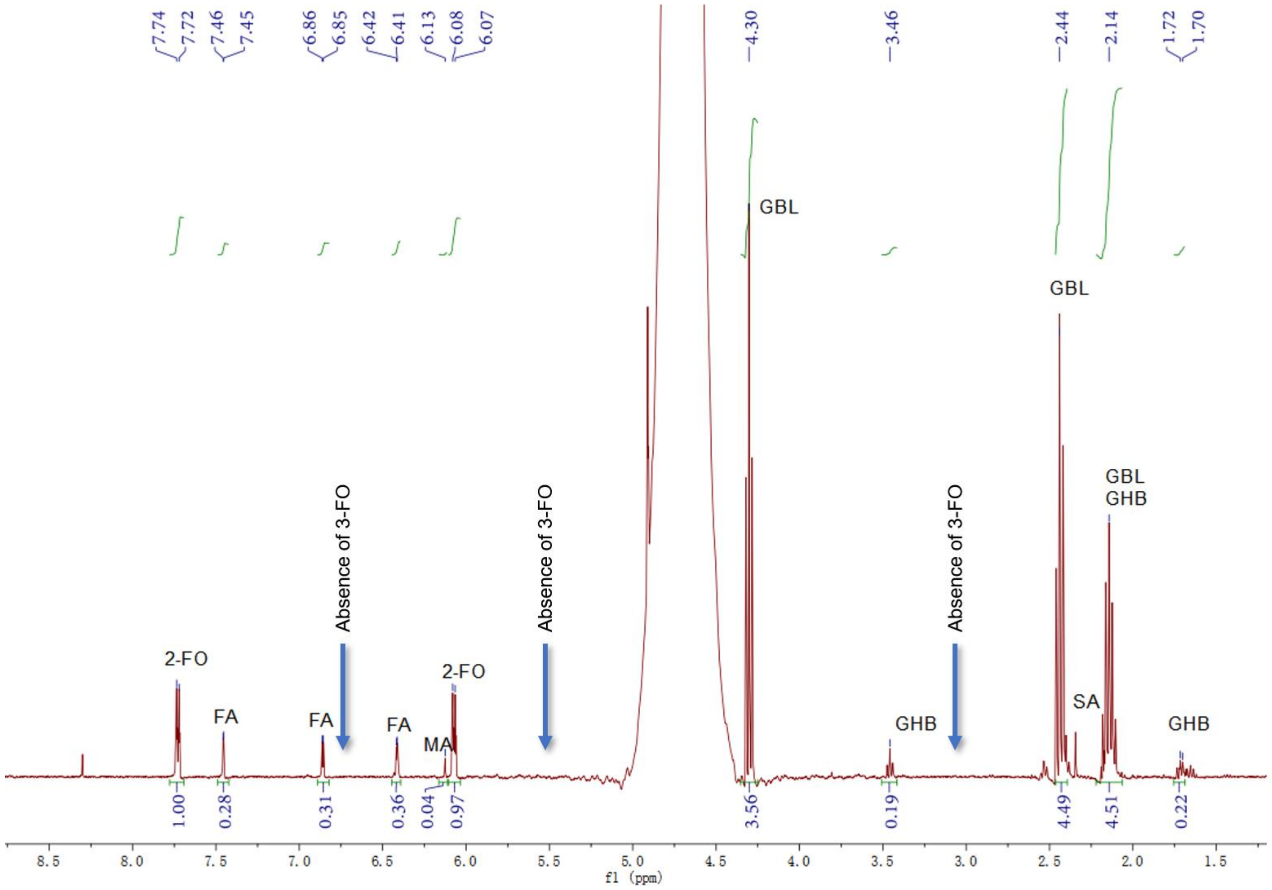

Fig. R11. Raw electrolysis ^{1}H NMR results in D_2O. One-pot electrochemical conversion of FA reaction passed charges 500 C. Reaction conditions: 100 mM FA in 20 mL pH=5.5 buffer; 80 °C; 2.0 $V_{Ag/AgCl}$; WE: Pt, CE: Ni. Note: GBL: Gamma-Butyrolactone; GHB: Gamma-Hydroxybutyrate; SA: Succinic acid.

4) The quoted selectivity should be based on conversion, not just detected products, as it makes it sound higher than it really is. All run data should also be compiled in an SI table (showing FE, carbon balance, yield, reaction conditions etc). It is hard to synthesise all the information when relayed in paragraph form. Faradaic efficiency and carbon balance should also be commented on in addition to the yield/selectivity of the products in the abstract.

Response: Thank you for this advice. We changed the selectivity calculation equation based on conversion and added the run data in Table S2 in the revised manuscript and SI file. FE and carbon balance were also added to the abstract.

$$Sel._x\ (\%) = \frac{mol_x}{mol_{reactant\ init.} - mol_{reactant}} \times 100\% \quad (3)$$

5) Some comparisons of OER onset are made with organic present and absent (Fig S5 and related comments). In the presence of organic, how is OER distinguished from the organic oxidation when only current is measured?

Response: Thank you for this question. We agree that in the presence of an organic substrate, part of the OER onset current is convoluted with organic oxidation. However, the Faradaic current resulted in organic oxidation is negligible compared to the OER because of the rapid scan rate of 50 mV s^{-1}, and the mass transport of fresh reductant, namely the FA, is relatively slow in the non-stirred environment of LSV experiments. Therefore, the observed sharp rise in current is OER. A comparable example measuring the OER onset potential change due to organic adsorptive inhibition (Fig. S8) is also commonly seen in the Kolbe electrolysis study, where volatile fatty acids adsorption shifted the OER onset potentials to more positive values.[6]

6) The formation of a furan "radical" is discussed, but this will not really be a radical if it is adsorbed to the surface. I would also change the phrasing to say this species is hypothesised — while the comments are plausible, there is no direct observation of this species.

Response: Thanks for your suggestion. It has been mentioned in some reported literature that the oxidation of FA forms free radicals through decarboxylation. We thus conducted EPR to monitor the formation of any radical species and captured them using 5,5-dimethyl-1-pyrroline *N*-oxide (DMPO) (Fig. R12), and added the following sentences to the revised manuscript.

We first verified the formation of the furan radical in our ECO system through electron paramagnetic resonance (EPR) spectroscopy. The results revealed the formation of a carbon-centred radical adduct 5,5-dimethyl-1-pyrroline *N*-oxide (DMPO-R; A_N = 15.3 G and A_H = 24.7 G) in the presence of FA (Fig. 1d),[12,13] providing strong evidence for the presence of the furanic radical. However, in the absence of FA, EPR showed only the DMPO-OH adduct (A_N = 14.9 G and A_H = 14.9 G) under the same reaction conditions. These results suggest that after decarboxylation, the furan radical combines with the OH radical on the Pt surface to yield hydroxyfuran, which subsequently undergoes tautomerisation to form 2-FO.

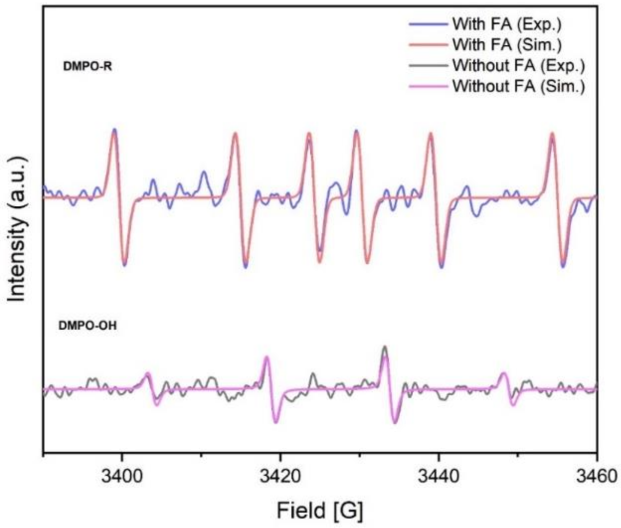

Fig. R12 (Fig. 1d). EPR study using DMPO. Reaction conditions: 20 mM FA with 400 mM DMPO in pH 5.5 buffer, 2.0 $V_{Ag/AgCl}$, 80 °C, WE: Pt, CE: Pt.

7) Comments around page 18 were confusing with regard to the experiments to evaluate inertness of electrodes toward reacting with the wrong species — for example referring to positive voltages that cause HER (when it is meant that these push the cathode toward a more negative voltage to match the current, and that causes HER). Given my comment #1 about showing plain CV's in wider voltage ranges, that whole discussion may likely change anyway.

Response: Thanks for your suggestion. We hope we have understood the reviewer correctly on this suggestion. We have edited the corresponding paragraph to clarify our message, and the paragraph has been moved to SI under Fig. S12. Specifically, we added "*After that, at +2.2 V, the corresponding cathodic working potential became too high that favoured HER instead of ECH of 2-FO to GBL, and thus the GBL yield and FE began to decline.*" As the reviewer suggested in the first comment, we have also conducted a plain CV in wider voltage range, showing the "wrong reactants" were electrochemically inactive towards the wrong electrode. The CV graphs are enclosed with the first comment.

8) There are no error bars or reproduction of even the optimised conditions. How dependable are the yields, FE, CB etc?

Response: Thank you for the advice. We repeated all the experiments in triplicate and added the error bars on each figure.

9) It was stated that Pt was chosen because it is stable. It was also stated that polymers likely accumulate on the surface, meaning that the metal itself may not be very important. Do other stable electrodes like Au give different results?

Response: Thanks for this advice. We also tried Au as anode at the same reaction conditions. We would also like to take the opportunity to clarify why Pt has great activity and selectivity in the oxidation of FA to 2-FO.

Here, we define selectivity as the production of 2-FO instead of other over-oxidised or ring-opening products, such as maleic acid (MA), succinic acid (SA), or furanic polymer. Activity is defined as the catalytic performance of electrocatalytic oxidised FA.

a) Pt's high selectivity in producing 2-FO, contributed by temperature

The high selectivity observed from Pt (oxidising FA to 2-FO) is actually due to the reaction condition at 80 °C and mildly acidic condition. In our reaction, FA undergoes electrocatalytic oxidative decarboxylation to yield a furan radical intermediate. The 80 °C reaction temperature promotes its thermal desorption from the electrode surface, allowing it to react with the water to yield 2-FO (Fig. R14c). In our revised manuscript, we have added an EPR study (Fig. 1d) that captured the presence of a carbon-centred radical species, which we deduced to be the furan radical. At low temperatures, e.g., 20 °C, the furan radical retained well on the electrode and polymerised (Fig. R14b), and thus produced more polymer on the Pt surface, which was observed in the ex-situ ATR-FTIR study (Fig. 1c).

b) Pt's high selectivity in producing 2-FO, contributed by pH

The mildly acidic environment at pH 5.5 was also important to enhance selectivity, as it promotes the deprotonation of FA, which can enhance anodic adsorption. The adsorption mode was supported by in-situ FTIR analysis and DFT calculations. [1,2] At pH 1, the conversion of FA reached 100% but the 2-FO yield decreased to 28.3% ± 1.8% and the HFO yield increased to 14.7% ± 2.6% (from 11.6% ± 1.3% at pH 2) (Table R3). The decreased yield of 2-FO may be attributable to weak furan ring-directed adsorption of FA, which promoted its overoxidation and mineralisation (Fig. R14a). This would have increased the HFO yield and the oxidative mineralising activity, as indicated by the low carbon balance (CB). To solve this problem, we used a pH of 5.5, at which FA was nearly entirely deprotonated, as indicated by an ultraviolet-visible spectroscopy analysis (Fig. S5). At pH 5.5, the CB was 89.0% ± 1.6%, whereas at pH 1, it was only 46.1% ± 3.1% (Table R3). The carboxylate form of FA, i.e., furoate anion, tended to adsorb in a bidentate manner and upright orientation onto the Pt surface (Fig. R14c),[1,2] and promoted the ECO of the carboxyl group. The linear sweep voltammetry (LSV) analyses show a greater current density difference (with and without FA) at pH 5.5 than at pH 1, indicating a stronger furoate inhibition on Pt at pH 5.5, and thus confirming the enhancement of surface adsorption of the furoate anions at greater pH. (Fig. R15).

Table R3. (Table S1) Electrocatalytic oxidation of FA

Entry	WE	pH	Conversion (%)	Yield of products (%)			Sel of 2-FO (%)	CB (%)
				HFO	MA	2-FO		
1	Pt	1	100 ± 0.0	14.7 ± 2.6	3.2 ± 1.3	28.3 ± 1.8	28.3 ± 1.8	46.1 ± 3.1
2	Pt	5.5	88.9 ± 3.0	1.3 ± 0.5	3.0 ± 0.5	74.8 ± 4.4	84.2 ± 2.4	89.0 ± 1.6
3	Au	5.5	8.4 ± 3.6	0.8 ± 0.6	0.4 ± 0.2	6.5 ± 2.7	77.3 ± 4.0	92.2 ± 4.1

Reaction conditions: 10 mM FA in 10 mL of 0.5 M buffer at +1.8 $V_{Ag/AgCl}$ referenced to the WE (working anode) paired with a Pt counter electrode at 80 °C.

c) 2-FO production activity contributed by metal catalyst

In terms of activity, we compared the electrocatalytic oxidation performance of FA between Pt and Au. We found that using gold (Au) electrodes afforded a poor yield of 2-FO (6.5% ± 2.7%) (Table R3). Density functional theory (DFT) calculations suggested that the Au surface was more susceptible than the Pt surface to being ‘poisoned’ by upright furoate, thereby hindering furoate decarboxylation or oxidation steps.[3]

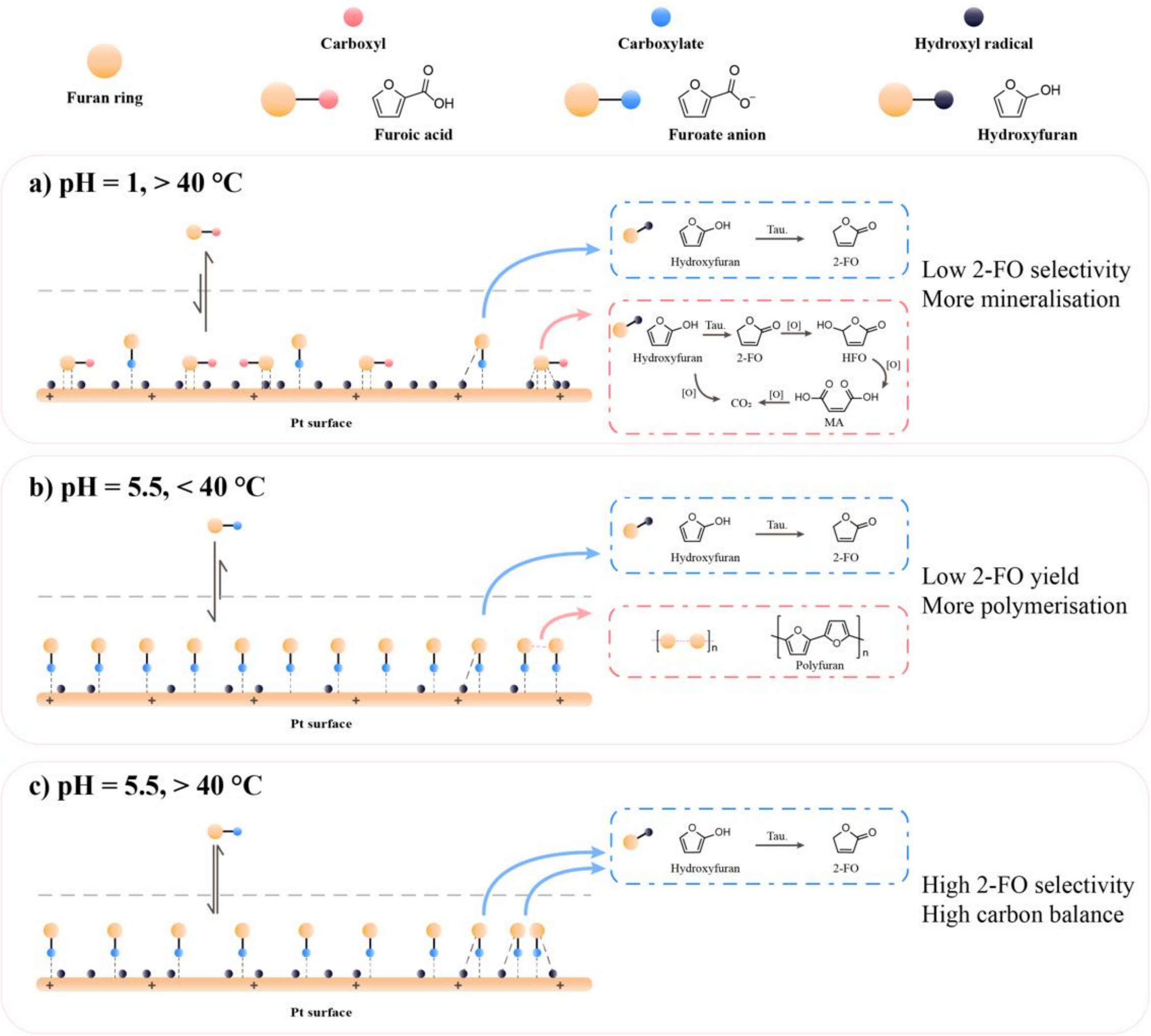

Fig. R14 (Fig. 2). Various anodic interface events at pHs 1 and 5.5 above and below 40 °C, respectively

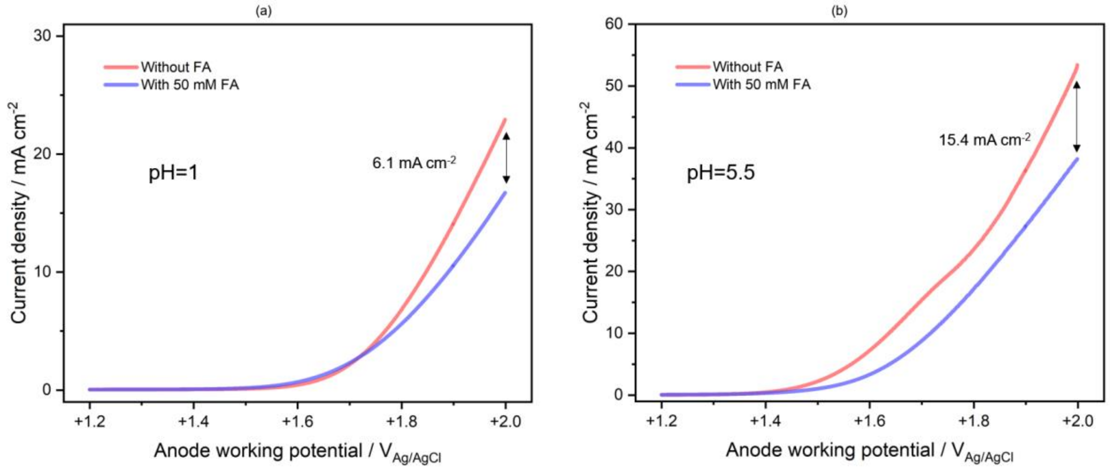

Fig. R15 (Fig. S6). LSV study at pH 1 and pH 5.5 with or without 50 mM FA. Reaction conditions: Scan rate 50 mV/s; 80 °C; WE: Pt; CE: Pt.

10) Most biomass processing routes are going to lead to furfural (FF) as a starting point. It was found that the reactor concept in this paper doesn't work well with FF because it is too reactive with the cathode, and thus the authors start with FA instead. It is implied that pre-oxidation FF->FA will be very easy, but I think this needs more discussion and qualification about what realistic rates and selectivities would be achievable, and how the whole path would be integrated as a process.

Response: Thank you for this suggestion. The oxidation of FF to FA has been investigated by using various methodologies, including thermal catalytic, electrocatalytic, and biocatalytic methods. Many of those methods are highly efficient and can be compatible with our FA to GBL one-pot synthesis. We included some of the selected studies in the introduction and towards the conclusion of the revised manuscript. We also summarised the additional examples in Table R4 in the SI.

In the introduction, we added the following paragraph: [line 96 – 105]

However, FA does not exhibit these problems and can be readily obtained from FAL via various mild methodologies. For instance, SiO_2-Co(acac)$_2$ catalysed the conversion of FAL into FA under solvent-free conditions, yielding an 85% isolated yield of FA within 5 h at 50 °C.[14] The solvent-free and heterogeneous nature of this method allowed the facile separation of FA. FA was also produced electrochemically from FAL in a 97% yield, with NiFe-1 as the anode and at 756 mA cm^{-2}, a current density that is compatible with industrial-scale processes.[15] Biocatalytic methods can be employed; for example, *Pseudomonas putida* KT2440 converted FAL into FA in over 97% yield at pH 6 and 30 °C over 2 h.[16] Additional examples of FAL-to-FA conversions are presented in Table S3.

Towards the conclusion, we added the following paragraph: [line 393 – 401]

Nevertheless, our one-pot FA-to-GBL method generated highly pure GBL at a rate compatible with those of existing FAL-to-FA technologies. Based on the conversion rate, our scaled-up reaction consumed 5.6 g of FA per 24 h, which is equivalent to 2083.3 µmol/h. A thermal catalytic method using MnO_2@CeO_2 under 8 bar O_2 at 130 °C converted FAL to FA at a rate of 750 µmol/h.[17] Biocatalytic methods could also provide high FAL to FA conversion rates, such as 2425 µmol/h.[16] Our one-pot FA-to-GBL electrosynthesis shows good compatibility in reaction rates with some of the existing FAL-to-FA technologies, which will allow their integrations with our synthesis of GBL.

In the SI, we added the following table:

Table R4 (Table S3). Comparison of different systems for oxidation of furfural to produce furoic acid.

Entry	Catalyst	Reaction conditions	Performance	Ref.
1	SiO_2-Co(acac)$_2$	Solvent-free, 50 °C, air, 5 h	85% isolated FA yield, 170 µmol/h	14
2	BTIC	125 mM FAL, DMSO with DBU, 40 °C, 0.1 MPa O_2, 4 h	> 99% FA yield, 62.5 µmol/h	18
3	MnO_2@CeO_2	300 mM FAL, H_2O, 130 °C, 8 bar O_2, 4 h	96% FA yield, 750 µmol/h	17
4	$Fe^{III}Mo_6$	750 mM FAL, 0.75 M $NaCO_3$, 50 °C, 1 atm O_2, 8 h	97% isolated FA yield, 182 µmol/h	19

5	NiFe-1	100 mM FAL, 1.478 V vs. RHE, 1 M KOH	97% FA yield, 96% FE, 756 mA cm^{-2}	[20]
6	NiMoP/NF	100 mM FAL, 1.46 V vs. RHE, 1 M NaOH	99.2% FA yield, 96.7% FE, 500 mA cm^{-2}	[21]
7	Ni_xSe_y–NiFe LDH@NF	20 mM FAL, 1.423 V vs. RHE, 1 M KOH	99.7% FA yield, 99.5% FE, ~ 100 mA cm^{-2}	[22]
8	Ag_2O@Ni foam	530 mM FAL, 1.95 V OCV, 1 M KOH	90% FA yield, 94% FE, 400 mA cm^{-2}	[23]
9	Comamonas testosteroni SC1588	50 mM FAL, 30 °C, pH 7 buffer, 10 h	96% FA yield, 20 μmol/h	[24]
10	*P. putida* KT2440	204 mM FAL, 30 °C, pH 6 buffer, 3 h	>97 FA yield, 2425 μmol/h	[16]

Reference

1 Hasse, J. C., Agrawal, N., Janik, M. J. & Holewinski, A. ATR-SEIRAS Investigation of the Electro-oxidation Mechanism of Biomass-Derived C5 Furanics on Platinum Electrodes. *The Journal of Physical Chemistry C* **126**, 7054-7065, doi:10.1021/acs.jpcc.2c01259 (2022).

2 Gong, L., Agrawal, N., Roman, A., Holewinski, A. & Janik, M. J. Density functional theory study of furfural electrochemical oxidation on the Pt (1 1 1) surface. *Journal of Catalysis* **373**, 322-335, doi:10.1016/j.jcat.2019.04.012 (2019).

3 Román, A. M. *et al.* Electro-oxidation of furfural on gold is limited by furoate self-assembly. *Journal of Catalysis* **391**, 327-335, doi:10.1016/j.jcat.2020.08.034 (2020).

4 A. Ruban, B. Hammer, P. Stoltze, H.L. Skriver & Nørskov, J. K. Surface electronic structure and reactivity of transition and noble metals. *Journal of Molecular Catalysis A: Chemical* **115**, 421-429, doi:https://doi.org/10.1016/S1381-1169(96)00348-2 (1997).

5 Delbecq F. & P., S. Competitive C 璡 C and C 璡 O Adsorption of α-β-Unsaturated Aldehydes on Pt and Pd Surfaces in Relation with the Selectivity of Hydrogenation Reactions: A Theoretical Approach. *Journal of Catalysis* **152**, 217-236, doi:https://doi.org/10.1006/jcat.1995.1077 (1995).

6 Fry, A. J. *Synthetic Organic Electrochemistry*. 2nd edn, 352 (1989).

7 Procida, A. & Honeychurch, K. C. Smartphone-based colorimetric determination of gamma-butyrolactone and gamma-hydroxybutyrate in alcoholic beverage samples. *J Forensic Sci* **67**, 1697-1703, doi:10.1111/1556-4029.15042 (2022).

8 Liu, H. & Li, W. Recent advances in paired electrolysis of biomass-derived compounds toward cogeneration of value-added chemicals and fuels. *Current Opinion in Electrochemistry* **30**, doi:10.1016/j.coelec.2021.100795 (2021).

9 Na, J. *et al.* General technoeconomic analysis for electrochemical coproduction coupling carbon dioxide reduction with organic oxidation. *Nat Commun* **10**, 5193, doi:10.1038/s41467-019-12744-y (2019).

10 Sbei, N., Hardwick, T. & Ahmed, N. Green Chemistry: Electrochemical Organic Transformations via Paired Electrolysis. *ACS Sustainable Chemistry & Engineering* **9**, 6148-6169, doi:10.1021/acssuschemeng.1c00665 (2021).

11 Cao, R., Liu, C. & Liu, L. A Convenient Synthesis of 2(5h)-Furanone. *Organic Preparations and Procedures International* **28**, 215-216, doi:10.1080/00304949609356524 (1996).

12 Shlotanl, M., Nagata, Y., Tasakl, M., Sohma, J. & Shida, T. Electron Spin Resonance Studies on Radical Cations of Flve-Membered Heteroaromatics. Furan, Thiophene, Pyrrole, and Related Compounds. *The Journal of Physical Chemistry A* **87**, 1170-1174, doi:10.1021/j100230a015 (1983).

13 Chen, L. *et al.* Accurate identification of radicals by in-situ electron paramagnetic resonance in ultraviolet-based homogenous advanced oxidation processes. *Water Res* **221**, 118747, doi:10.1016/j.watres.2022.118747 (2022).

14 Sodhi, R. K., Paul, S. & Clark, J. H. A comparative study of different metal acetylacetonates covalently anchored onto amine functionalised silica: a study of the oxidation of aldehydes and alcohols to corresponding acids in water. *Green Chemistry* **14**, doi:10.1039/c2gc35289c (2012).

15 Wang, C. *et al.* A Novel Electrode for Value-Generating Anode Reactions in Water Electrolyzers at Industrial Current Densities. *Angew Chem Int Ed Engl* **62**, e202215804, doi:10.1002/anie.202215804 (2023).

16 Zheng, Z., Xu, Q., Tan, H., Zhou, F. & Ouyang, J. Selective Biosynthesis of Furoic Acid From Furfural by Pseudomonas Putida and Identification of Molybdate Transporter Involvement in Furfural Oxidation. *Front Chem* **8**, 587456, doi:10.3389/fchem.2020.587456 (2020).

17 Nocito, F. *et al.* Selective Aerobic Oxidation of Furfural into Furoic Acid over a Highly Recyclable MnO2@CeO2 Core–Shell Oxide: The Role of the Morphology of the Catalyst. *ACS Sustainable Chemistry & Engineering* **10**, 8615-8623, doi:10.1021/acssuschemeng.2c02341 (2022).

18 Gupta, N. K., Fukuoka, A. & Nakajima, K. Metal-Free and Selective Oxidation of Furfural to Furoic Acid with an N-Heterocyclic Carbene Catalyst. *ACS Sustainable Chemistry & Engineering* **6**, 3434-3442, doi:10.1021/acssuschemeng.7b03681 (2018).

19 Yu, H. *et al.* An Efficient Iron(III)-Catalyzed Aerobic Oxidation of Aldehydes in Water for the Green Preparation of Carboxylic Acids. *Angew Chem Int Ed Engl* **56**, 3867-3871, doi: 10.1002/ange.201612225 (2016).

20 Wang, C. *et al.* A Novel Electrode for Value-Generating Anode Reactions in Water Electrolyzers at Industrial Current Densities. *Angew Chem Int Ed Engl* **62**, doi:10.1002/ange.202215804 (2023).

21 Zeng, Z., Wu, S., Huang, X. & Wei, Z. Electrochemical Oxidation of Furfural on NiMoP/NF: Boosting Current Density with Enhanced Adsorption of Oxygenates. *Small*, e2305462, doi:10.1002/smll.202305462 (2023).

22 Zhong, Y. *et al.* Grass-like NixSey nanowire arrays shelled with NiFe LDH nanosheets as a 3D hierarchical core–shell electrocatalyst for efficient upgrading of biomass-derived 5-hydroxymethylfurfural and furfural. *Catalysis Science & Technology* **12**, 201-211, doi:10.1039/d1cy01816g (2022).

23 Ouyang, D., Gao, D., Hong, J., Jiang, Z. & Zhao, X. Promoting and controlling electron transfer of furfural oxidation efficiently harvest electricity, furoic acid, hydrogen gas and hydrogen peroxide. *Journal of Energy Chemistry* **79**, 135-147, doi:https://doi.org/10.1016/j.jechem.2022.10.036 (2023).

24 Shi, S.-S., Zhang, X.-Y., Zong, M.-H., Wang, C.-F. & Li, N. Selective synthesis of 2-furoic acid and 5-hydroxymethyl-2-furancarboxylic acid from bio-based furans by recombinant Escherichia coli cells. *Molecular Catalysis* **469**, 68-74, doi:10.1016/j.mcat.2019.03.006 (2019).

REVIEWERS' COMMENTS

Reviewer #1 (Remarks to the Author):

The authors have addressed the concerns raised by the reviewers. I recommend this work to be published in Nature communications.

Reviewer #3 (Remarks to the Author):

I have read through all responses and believe the reviewer comments have been addressed sufficiently. I recommend publication.
